# First-principles prediction of high oxygen-ion conductivity in trilanthanide gallates Ln_3_GaO_6_

**DOI:** 10.1080/14686996.2019.1578183

**Published:** 2019-02-06

**Authors:** Joohwi Lee, Nobuko Ohba, Ryoji Asahi

**Affiliations:** Toyota Central R&D Laboratories, Inc., Nagakute, Japan

**Keywords:** Oxygen-ion conductor, Ln_3_GaO_6_, lanthanide gallate, first-principles material design, 50 Energy Materials, 207 Fuel cells / Batteries / Super capacitors, 401 1st principle calculations, 107 Glass and ceramic materials, 404 Materials informatics / Genomics

## Abstract

We systematically investigated trilanthanide gallates (Ln_3_GaO_6_) with the space group *Cmc*2_1_ as oxygen-ion conductors using first-principles calculations. Six Ln_3_GaO_6_ (Ln = Nd, Gd, Tb, Ho, Dy, or Er) are both energetically and dynamically stable among 15 Ln_3_GaO_6_ compounds, which is consistent with previous experimental studies reporting successful syntheses of single phases. La_3_GaO_6_ and Lu_3_GaO_6_ may be metastable despite a slightly higher energy than those of competing reference states, as phonon calculations predict them to be dynamically stable. The formation and the migration barrier energies of an oxygen vacancy (*V*_O_) suggest that eight Ln_3_GaO_6_ (Ln = La, Nd, Gd, Tb, Ho, Dy, Er, or Lu) can act as oxygen-ion conductors based on *V*_O_. Ga plays a role of decreasing the distances between the oxygen sites of Ln_3_GaO_6_ compared with those of Ln_2_O_3_ so that a *V*_O_ migrates easier with a reduced migration barrier energy. Larger oxygen-ion diffusivities and lower migration barrier energies of *V*_O_ for the eight Ln_3_GaO_6_ are obtained for smaller atomic numbers of Ln having larger radii of Ln^3+^. Their oxygen-ion conductivities at 1000 K are predicted to have a similar order of magnitude to that of yttria-stabilized zirconia.

## Graphical abstract

**Figure:**
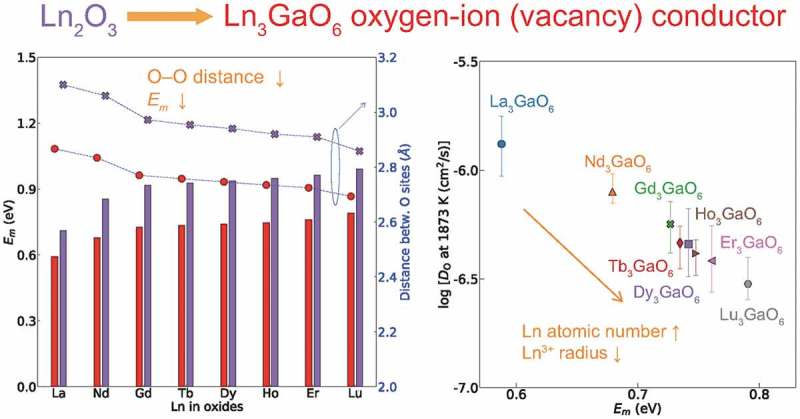

## 1. Introduction

Oxygen-ion conductors with high oxygen-ion conductivities (*σ*_O_) have been developed for important applications [1,2], such as electrolytes of solid oxide fuel cells (SOFC), oxygen separation membranes, and gas sensors. Currently, yttria-stabilized zirconia (YSZ) is widely used because of its advantages, such as abundance, chemical stability, non-toxicity, and low cost. This material is known to exhibit a *σ*_O_ of ~10^−2^ S/cm at a high temperature of 1000 K [2,3]. For useful industrial applications, it is necessary to be further improved to have similar *σ*_O_ values at lower temperatures or a higher *σ*_O_ at a similar temperature. Indeed, there are several oxides such as Gd-doped CeO_2_ (GDC) [4] and pure or Er-doped *δ*-phase of Bi_2_O_3_ [5,6], which have been reported to have higher *σ*_O_ at the same temperature than YSZ. However, there have been great efforts for the development of new oxygen-ion conductors that can substitute for YSZ with enough merits in practice.

Lanthanide gallates (Ln–Ga–O) have also been investigated as oxygen ionic conductors. Actually, Sr- and Mg-doped LaGaO_3_ (LSGM) [7–11] with a perovskite structure have been widely developed as an electrolyte for intermediate temperature (~500–750 °C) [12] SOFCs because their *σ*_O_ is higher than that of YSZ at this temperature. In addition, trilanthanide galla tes in another composition ratio (Ln_3_GaO_6_) have also been introduced as oxygen-ion conductors. Purohit et al. [13] reported that pure and Sr- or Ca-doped Nd_3_GaO_6_ achieved a *σ*_O_ of 2.7 × 10^−4^ and 0.7 × 10^−2^ S/cm at 800 °C, respectively. Iakovleva et al. [14] reported that a Sr-doped Gd_3_GaO_6_ achieved a total conductivity of 0.9 × 10^−2^ S/cm (with *σ*_O_ of 0.3 × 10^−2^ S/cm and the remaining conductivity likely originating from protons) at 800 °C. Their *σ*_O_ values are not significantly different from that of YSZ at ~1000 K. Some Ln_3_GaO_6_ (Ln = Nd, Sm, Eu, Gd, Tb, Dy, Ho, or Er) were synthesized as a single phase with the space group *Cmc*2_1_ [15–17]. The abovementioned experimental reports suggest that other Ln_3_GaO_6_ compounds may show similar (or higher) *σ*_O_ values.

In contrast to experimental reports on Ln_3_GaO_6_, first-principles calculations on these ternary oxides are very rare. However, for binary lanthanide sesquioxides (Ln_2_O_3_) [18–21], the locations of the *f*-levels inside the O 2*p*–Ln 5*d* band gap (*E_g_*) have been predicted using first-principles calculations (see Section 3.2). Whether the localized *f*-levels exist inside or outside of *E_g_*(O 2*p*–Ln 5*d*) depending on the type of Ln (number of *f*-electrons) changes the characteristics of the highest occupied molecular orbital (HOMO) and the lowest unoccupied molecular orbital (LUMO), and the *E_g_* value. Therefore, it is useful to investigate the electronic structures of Ln_3_GaO_6_ by the first-principles calculations.

In this study, we used the systematic first-principles calculations to determine the structural and energetic properties based on the crystal and electronic structures of Ln_3_GaO_6_ with all types of Ln. We classified the type of Ln_3_GaO_6_ according to the location of *f*-levels in the band gap. Then, for the Ln_3_GaO_6_ type, whose electronic structures are less affected by the *f*-levels, we further investigated the energetic and dynamical stabilities, and the properties related to *σ*_O_, namely, the formation energy (*E_v_*) and migration barrier energy (*E_m_*) of the oxygen vacancy (*V*_O_). Finally, we compared the computed oxygen-ion diffusivity (*D*_O_) among the Ln_3_GaO_6_ types including the *V*_O_ by the first-principles molecular dynamics (FPMD) and predicted their *σ*_O_ values.

## 2. Methodology

### 2.1. Crystal structure

**Figure 1.**
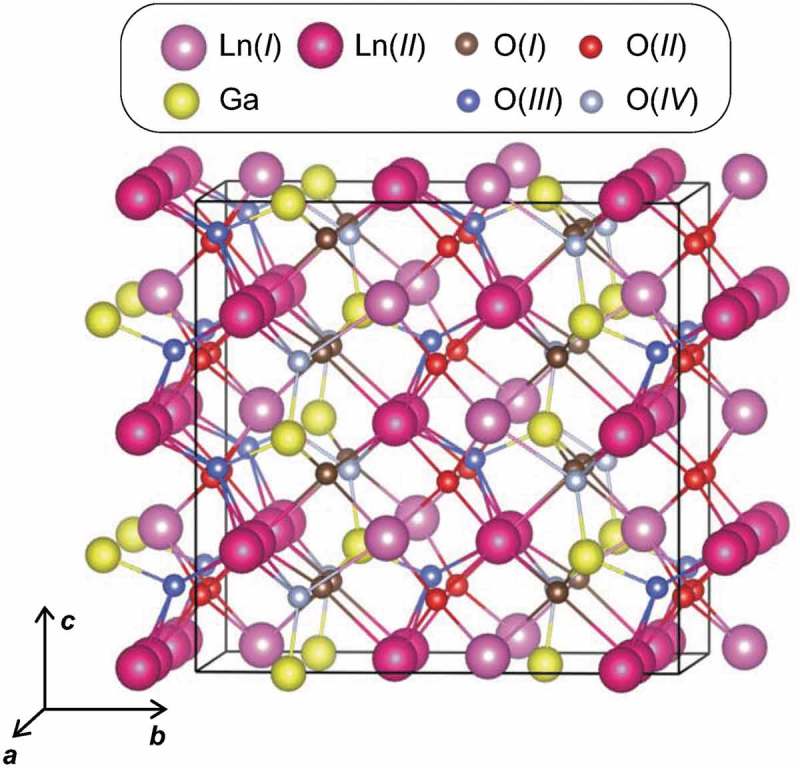
Crystal structure of Ln_3_GaO_6_ with space group *Cmc*2_1_. The supercell (1 × 1 × 2 conventional orthorhombic unit cell) with 24 Ln, 8 Ga, and 48 O atoms is shown. Different types of Ln and O sites are presented by different colors.

Figure 1 shows the crystal structure of Ln_3_GaO_6_ in the orthorhombic crystal system with the space group *Cmc*2_1_. This crystal structure consists of two, one, and four types of Ln, Ga, and O sites, respectively. The Wyckoff letters with the multiplicity of La(*I*), La(*II*), Ga, O(*I*), O(*II*), O(*III*), and O(*IV*) are 8*a*, 16*b*, 8*a*, 16*b*, 16*b*, 8*a*, and 8*a*, respectively, when we consider them in the crystal structure shown in Figure 1. The coordination numbers (CNs) of Ln [both of La(*I*) and La(*II*)] and Ga with respect to O are seven and four, respectively. Note that the CN of Ln in the binary Ln_2_O_3_ (both of cubic and hexagonal crystal systems) is six. O(*I*), O(*II*), O(*III*), and O(*IV*) have three, four, four, and three chemical bonds with Ln, respectively. In addition, O(*I*), O(*III*), and O(*IV*) have one chemical bond with Ga, whereas O(*II*) has no chemical bond with Ga, but only with Ln. In total, we obtain 15 types of Ln_3_GaO_6_ (Ln = La, Ce, Pr, Nd, Pm, Sm, Eu, Gd, Tb, Dy, Ho, Er, Tm, Yb, or Lu) in this crystal structure.

### 2.2. First-principles calculations

All first-principles calculations were performed using the project augmented wave (PAW) [22,23] method implemented in the Vienna *Ab-initio* Simulation Package (VASP) [24,25] within the framework of a generalized gradient approximation (GGA) of Perdew–Burke–Ernzerhof (PBE) form [26]. As the valence electrons, 5*p*, 5*d*, and 6*s* for Ln (5*s* is also included for La, Ce, Pr, Nd, Pm, and Sm), 3*d*, 4*s*, and 4*p* for Ga, and 2*s* and 2*p* for O were considered. In addition, on-site Coulomb interaction [27] with an effective U−J of 5 eV (GGA+U) for the *f*-orbitals of Ln was partially employed in the calculations. The value of U−J was selected by referring to previous theoretical calculations [19,28–30] on Ln_2_O_3_, which suggested an adequate range of the value as 5–7 eV with crosschecks on experimental reports. For the GGA+U method, 4*f*, 5*s*, 5*p*, 5*d*, and 6*s* electrons of Ln were also considered as the valence electrons. Henceforth, we refer to the former and latter methods as the GGA/w.o.f (without *f*-valences but in the frozen core) and GGA+U/w.f methods (with *f*-valences), respectively.

The structural relaxations of primitive unit cells with the associated changes in lattice constants and atomic coordinates were performed until the interatomic force on each atom was reduced to within 0.005 eV/Å. The cutoff energy was set as 500 eV. Brillouin zone was sampled by using Γ-centered 4 × 4 × 4 meshes. Gaussian smearing for the Brillouin zone integrations was employed with widths of 0.1 eV. Electronic spin polarizations were basically turned on except for the case where the up spin and down spin were confirmed to be compensated by each other.

We also computed *E_v_* and *E_m_* because their low values are favorable for achieving a higher *σ*_O_ [31,32]. The *E_v_* values were computed using the supercell method [33] as follows:

(1) $$E_{v}^{q}=E\left(Ln_{3}GaO_{6}:V_{O}^{q}\right)-E\left(Ln_{3}GaO_{6}\right)+\mu_{O}+q\left(E_{HOMO}+E_{Fermi}\right)+\Delta E_{LZ}$$

where *E*(Ln_3_GaO_6_:*V*_O_ *^q^*) is the energy of a supercell including a *V*_O_, *E*(Ln_3_GaO_6_) is the energy of a supercell of pure Ln_3_GaO_6_, *μ*_O_ is the chemical potential of O, *q* is the charge of *V*_O_, *E*_HOMO_ is the eigenvalue of the HOMO level mainly formed by O 2*p, E_Fermi_* is the Fermi level as a variable, and Δ*E*_LZ_ is the correction term suggested by Lany and Zunger [34,35] to compensate for the image charge interactions between supercells for the charged *V*_O_. The calculations for the supercell with a *V*_O_ were performed until the interatomic forces on each atom were reduced to within 0.02 eV/Å for fixed lattice constants. The sizes of the supercells (80 atoms) were set as 1 × 1 × 2 orthorhombic conventional unit cells as shown in Figure 1. The Γ-centered 2 × 2 × 2 meshes were used for the ***k***-space sampling. The transformations between primitive and orthorhombic conventional unit cells were performed by SPGLIB implemented in PHONOPY [36,37]. The dielectric constants for the correction term of the image charge interaction were obtained using primitive cells with the density functional perturbation theory (DFPT) [38]. When *V*_O_ are charged, neutralizing background charges were added.

The phonon calculations of Ln_3_GaO_6_ were performed by using PHONOPY [36,37]. To calculate the phonon frequencies, we combined the supercell approach and DFPT [38]. Force constants of the oxides were calculated by applying DFPT at the Γ point to the supercell model (the same size supercell with 80 atoms) of the oxides. Phonon frequencies were then calculated from the force constants. Ln_3_GaO_6_ compounds were judged to be dynamically stable if they did not have imaginary phonon frequencies in their phonon densities of states (DOSs).

The *E_m_* values were calculated using the climbing image nudged elastic band (CI-NEB) method [39,40] with three intermediate images. The CI-NEB calculations were performed until the forces decreased below 0.03 eV/Å with a spring constant of 5 eV/Å^2^ between the images. We employed a doubly charged oxygen vacancy (*V*_O_^2+^) for *E_m_* assuming that it was formed by the reduction in cation valence upon doping.

### 2.3. First-principles molecular dynamics

In order to investigate *D*_O_, we performed FPMD using the same size supercell as for the computations of *E_v_* and *E_m_* including one *V*_O_^2+^. For computational efficiency, the cutoff energy was decreased to 300 eV, only Γ-point was sampled for the Brillouin zone, and the valence electrons were considered as 5*p*, 5*d*, and 6*s* for Ln (5*s* is also included for La and Nd), 4*s* and 4*p* for Ga, and 2*s* and 2*p* for O. For O, a soft pseudopotential with a reduced maximum cutoff energy (288 eV) was employed. The GGA/w.o.*f* method was used without spin polarizations.

The *D* value of each chemical element at high temperatures can be obtained from the mean square displacement (MSD) after a long simulation time *t* by the following equation [41,42]:

(2) $$D\left(t\right)=\lim_{t\to \infty }\frac{MSD}{6t}$$

Here, MSD(*t*) can be obtained by

(3) $$MSD\left(t\right)=\frac{1}{n}\underset{i}{\sum }{\left|r_{i}\left(t\right)-r_{i}\left(0\right)\right|}^{2}$$

where *n* is the number of atoms of each chemical element, and *r_i_*(0) and *r_i_*(*t*) are the positions of the *i*th atom at the time origin and new time *t*, respectively. To improve the statistics, the MSD is smoothed by averaging MSD(*t, t*_0_) using multiple time origins *t*_0_ by Equation (4).

(4) $$MSD\left(t,t_{0}\right)=\frac{1}{n}\underset{i}{\sum }{\left|r_{i}\left(t+t_{0}\right)-r_{i}\left(t_{0}\right)\right|}^{2}$$

The interval of 2 *f*s for each cycle of atomic movements was used. For the first 1000 cycles, the temperature was elevated from 573 K to the target temperature of 1873 K. Then, FPMD calculations were performed on the basis of Nose thermostat [43,44] with a canonical ensemble for 150,000 cycles taking the average over time from 4 to 300 ps to obtain the MSD with enough movements of O atoms without considering the first 2000 cycles. *D* is averaged for three trials of FPMD, with different initial positions of *V*_O_^2+^ at O(*I*), O(*II*), and O(*III*) sites.

## 3. Results and discussion

### 3.1. Crystal structure

**Figure 2.**
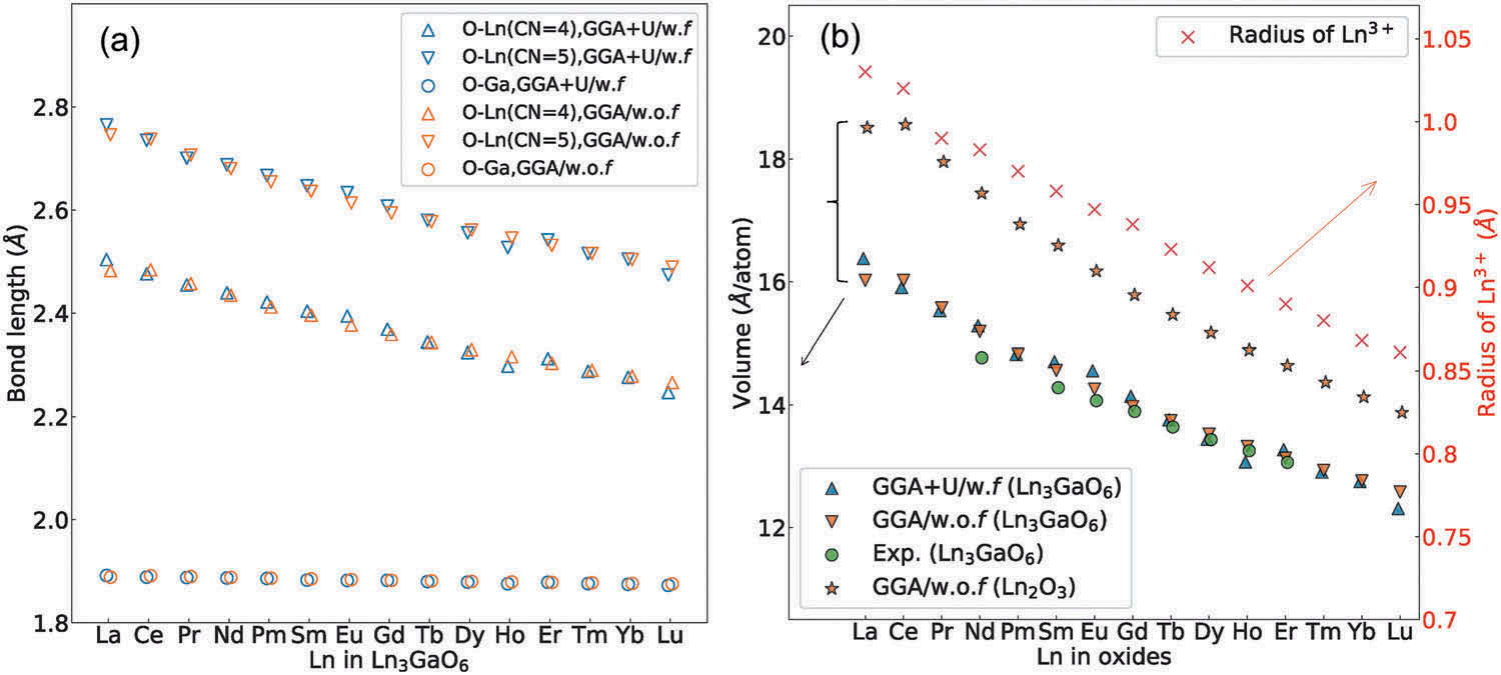
(a) Mean bond lengths of 15 Ln_3_GaO_6_ and (b) volume (eV/atom, left vertical axis) of 15 Ln_3_GaO_6_ and Ln_2_O_3_, and Ln^3+^ radius (right vertical axis). The experimental values of volume and the radius of Ln^3+^ are from Refs. [15] and [45], respectively.

Figure 2(a) shows the O–Ln and O–Ga bond lengths of the 15 types of Ln_3_GaO_6_ in this study. We separate the two types of O–Ln according to the CN of O. Calculated and experimental values [15], including Ln–O and Ga–O bond lengths, are provided in Supplementary Table S1. Note that when we obtain the mean bond lengths, the O–Ln bond lengths are counted as the distances between O and its three or four neighboring Ln, while the Ln–O bond lengths are counted as the distances between Ln and its seven neighboring O. We only considered the bond lengths among the distances between O and cations within a cutoff radius of 1.3 × the maximum peak obtained from the radial distribution functions (see Supplementary Figure S1). Our computational results obtained by both the GGA+U/w.*f* and GGA/w.o.*f* methods are consistent with the experimental reports. The computed bond lengths are slightly longer than the experimental bond lengths with an error of 3.5%. The differences of the bond lengths between the GGA+U/w.*f* and GGA/w.o.*f* methods are very small.

In general, the Ln–O bond lengths decrease with increasing atomic numbers of Ln. For the GGA+U/w.*f* method, the O–La (CN of O = 4) and O–La (CN of O = 5) bond lengths are longer than O–Lu (CN of O = 4) and O–Lu (CN of O = 5) bond lengths by 0.26 and 0.29 Å, respectively. However, the O–Ga bond lengths show a little variation with the atomic number of Ln. The O–Ga bond lengths of La_3_GaO_6_ and Lu_3_GaO_6_ differ from each other only by 0.02 Å.

Figure 2(b) shows the volumes of Ln_3_GaO_6_ and the radii of Ln^3+^ (*r*_Ln3+_). The calculated and experimental values [15] of volumes and lattice constants are mutually consistent, as shown in Supplementary Table S2.

As with the O–Ln bond lengths, in general, the volumes of Ln_3_GaO_6_ decrease with increasing atomic numbers of Ln. In addition, these values have a strong correlation with *r*_Ln3+_. Considering that the O–Ga bond lengths change little among Ln_3_GaO_6_, these results suggest that the size of Ln_3_GaO_6_ depends on *r*_Ln3+_.

In addition, we also compare the volumes of Ln_3_GaO_6_ and binary Ln_2_O_3_ (space group *Ia*-3 in the cubic crystal system) to determine the volume difference due to the existence of Ga as shown in Figure 2(b). As with Ln_3_GaO_6_, the decreasing tendency of the volume of Ln_2_O_3_ with increasing atomic number of Ln is found, which is consistent with a previous study by Petit et al. [18]. The volumes per atom of Ln_3_GaO_6_ are smaller than those of Ln_2_O_3_ by 9–15%. This may be ascribed to the considerably smaller ionic radius of Ga^3+^ (0.62 Å) than those of Ln^3+^ (0.86–1.03 Å) [45].

### 3.2. Electronic structure and location of f-levels

Before proceeding to our computational results of Ln_3_GaO_6_, it is worth reviewing previous studies on the locations of localized *f*-levels inside or outside of the *E_g_* of Ln_2_O_3_. Without *f*-levels, HOMO and LUMO are mainly generated by O 2*p* and Ln 5*d* orbitals, respectively. However, because the localized *f*-levels locate inside *E_g_*(O 2*p*–Ln 5*d*), the *E_g_* values and the characteristics of orbitals forming HOMO and LUMO strongly depend on Ln and its *f*-orbital filling. To correct the location of *f*-levels, Jiang et al. [19,20] employed GW-level calculations based on the wavefunctions of the LDA+U method and obtained similar *E_g_* values to experimental values. Gillen et al. [21] used hybrid functionals, namely, HSE03 and HSE06, and screened exchange local-density-approximations (sx-LDA). Altman et al. [46] observed the dependence of the location of *f*-levels on the type of Ln by X-ray absorption spectroscopy. These studies show a common trend: La_2_O_3_, Gd_2_O_3_, and Lu_2_O_3_, which have the earliest, the middle, and the last *f*-numbers of Ln, respectively, have less dependence on *E_g_*(O 2*p*–Ln 5*d*) for the *f*-levels because their *f*-levels are relatively far from the O 2*p*-HOMO and Ln 5*d*-LUMO. Ce_2_O_3_ and Tb_2_O_3_, which have one larger *f*-number of Ln than La_2_O_3_ and Gd_2_O_3_, respectively, have occupied *f*-levels that are located much higher than those of La_2_O_3_ and Gd_2_O_3_. Then, from Ce_2_O_3_ to Gd_2_O_3_, and from Tb_2_O_3_ to Lu_2_O_3_, the locations of the occupied and unoccupied *f*-levels monotonically get shifted to lower levels with increasing atomic number of Ln. In the result, Ce_2_O_3_ and Pr_2_O_3_ have *E_g_*(Ln 4*f*–Ln 5*d*) with the occupied *f*-levels located above the O 2*p* levels, whereas Ln_2_O_3_ (Ln = Pm, Sm, Eu, Tm, or Yb) have *E_g_*(O 2*p*–Ln 4*f*) with unoccupied *f*-levels located below Ln 5d levels. For the other Ln_2_O_3_, the locations of the *f*-levels are shallow or out of *E_g_*(O 2*p*–Ln 5*d*).

GW-level calculations can yield more accurate electronic structures; however, with current computational resources, it is not easy to systematically apply them to various materials that have large unit cells. Alternatively, we analyzed the location of the *f*-levels using two steps; first, we directly analyzed the *f*-levels using the projected electronic DOSs (PDOSs) by the GGA+U/w.*f* method. Then, we compared the *E_g_* values of the GGA/w.o.*f* and GGA+U/w.*f* methods.

**Figure 3.**
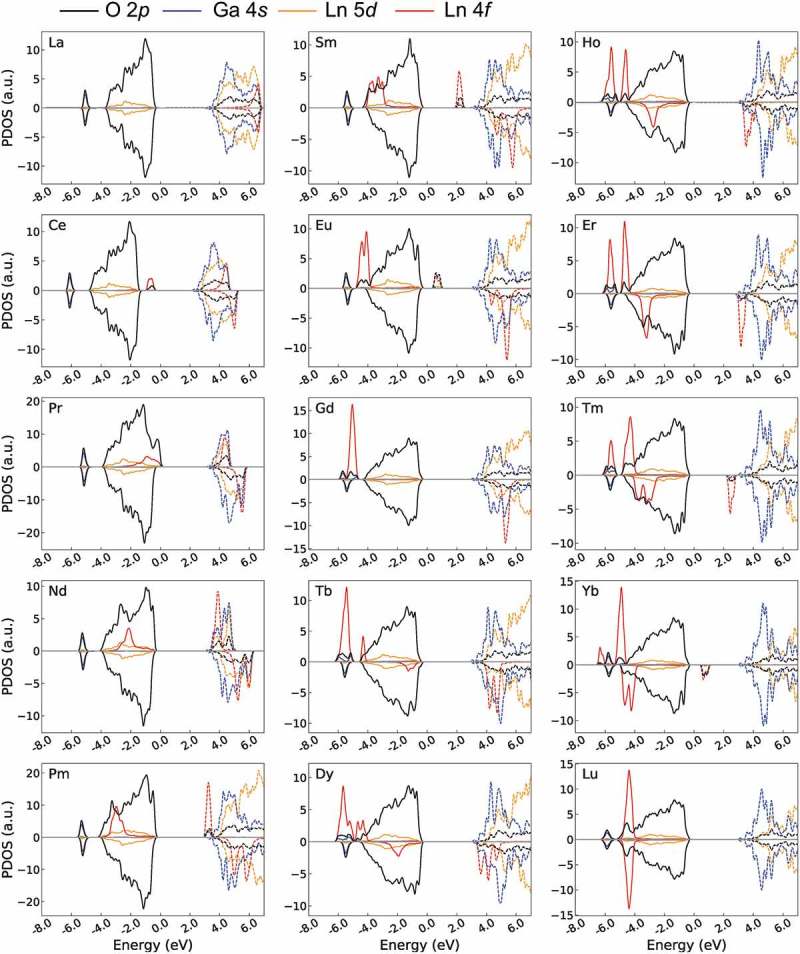
Electronic PDOS of 15 Ln_3_GaO_6_ obtained by the GGA+U/w.*f* method. Only Ln 4*f* (red) and 5*d* (yellow), Ga 4*s* (blue), O 2*p* (black) levels are drawn for simplicity. Energy is shifted by the computational Fermi level. Occupied and unoccupied levels are drawn as solid and dashed lines, respectively. Positive and negative values of PDOS denote the up-spin and down-spin states of electrons, respectively. For easier viewing, the unoccupied La 5*d*, Ga 4*s*, and O 2*p* levels are 2, 30, and 2 times magnified, respectively, whereas the Ln 4*f*-levels are 0.15 times decreased.

We analyzed whether the *f*-levels are formed inside *E_g_* using the PDOSs obtained by the GGA+U/w.*f* method as shown in Figure 3. Without the *f*-levels, the HOMO and LUMO are mainly generated by O 2*p* and Ga 4*s* orbitals, respectively, which differs from that of Ln_2_O_3_. Unoccupied Ln 5*d*-levels are slightly higher than the Ga 4*s*-LUMO (also see the PDOS obtained by the GGA/w.o.*f* method in Supplementary Figure S2).

For La_3_GaO_6_, the unoccupied and occupied *f*-levels are generated far from the O 2*p*-HOMO and Ga 4*s*-LUMO, respectively, and spin polarizations are almost compensated. For Ce_3_GaO_6_, the occupied *f*-levels at the up-spin state are clearly located over the O 2*p* and form the HOMO decreasing the *E_g_* value. Then, from Pr_3_GaO_6_ to Gd_3_GaO_6_, the occupied and unoccupied *f*-levels at the up-spin states move downward together with increasing atomic number of Ln. Gd_3_GaO_6_ has a clear *E_g_*(O 2*p*–Ga 4*s*) with the occupied *f*-levels at the up-spin state and unoccupied *f*-levels at the down-spin state far from the O 2*p*-HOMO and Ga 4*s*-LUMO, respectively. For Tb_3_GaO_3_6, the occupied *f*-level at the down-spin state is generated just below the O 2*p*-HOMO. Then, from Tb_3_GaO_6_ to Lu_3_GaO_6_, the occupied and unoccupied *f*-levels at the down-spin state move downward again with increasing atomic number of Ln. Finally, Lu_3_GaO_6_ has all occupied *f*-levels under O 2*p*-LUMO with compensated spin polarizations.

**Figure 4.**
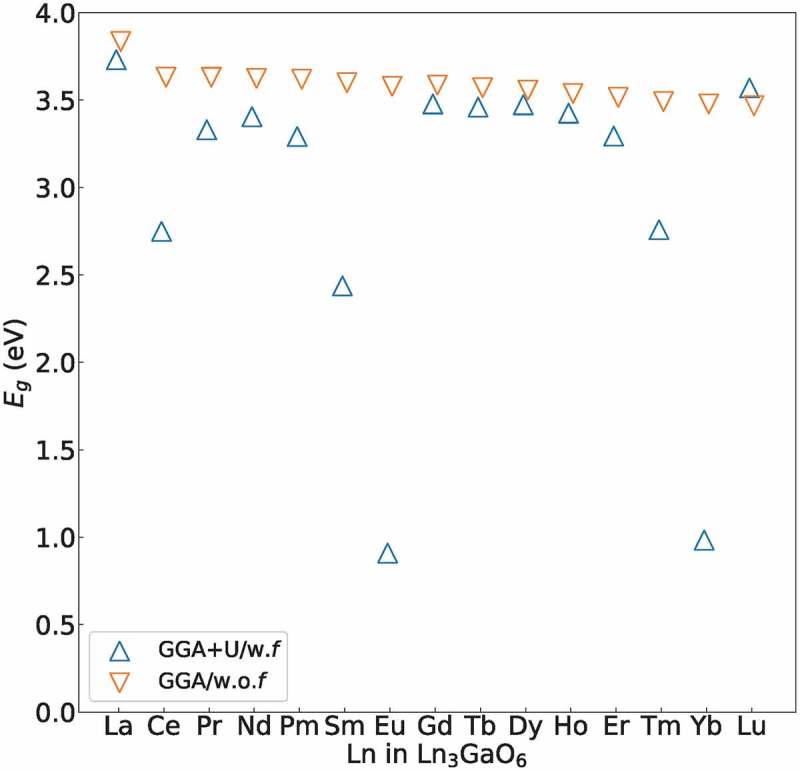
*E_g_* values of 15 Ln_3_GaO_6_ obtained by the GGA+U/w.*f* and GGA/w.o.*f* methods. The *E_g_* values of eight Ln_3_GaO_6_ (Ln = La, Nd, Gd, Tb, Ho, Dy, Er, or Lu) are not significantly different from each other because occupied or unoccupied *f*-levels are not formed deeply inside *E_g_*(O 2*p*–Ga 4*s*).

In addition, we compared the *E_g_* values obtained by the GGA+U/w.*f* and GGA/w.o.*f* methods. Figure 4 shows the *E_g_* values of the GGA+U/w.*f* and GGA/w.o.*f* methods. The *E_g_*(O 2*p*–Ga 4*s*) obtained by the GGA/w.o.*f* method are similar for all Ln_3_GaO_6_ with values of 3.47–3.83 eV. On the other hand, the *E_g_* values obtained by the GGA+U/w.*f* method significantly change depending on the types of Ln because of the location of *f*-levels. As suggested by Lal and Gaur [47], we can classify Ln_3_GaO_6_ into four groups according to the types of orbitals that form the LUMO and HOMO. We also consider the numerical difference of *E_g_* between the GGA+U/w.*f* and GGA/w.o.*f* methods (|Δ*E_g_*|).

**Figure 5.**
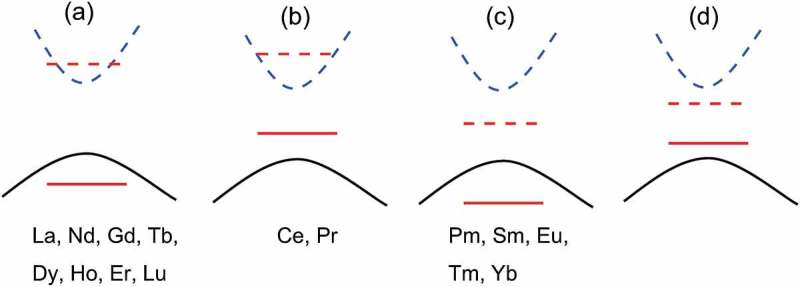
Schematic for the classification of Ln_3_GaO_6_ according to the location of occupied (red solid line) and unoccupied (red dashed line) *f*-level of Ln inside *E_g_*(O 2*p*–Ga 4*s*). Black solid and blue dashed curves denote the HOMO (O 2*p*) and LUMO (Ga 4*s*), respectively. (a) The first group of Ln_3_GaO_6_ has *E_g_*(O 2*p*–Ga 4*s*) less affected by the location of *f*-levels of Ln. (b) The second group of Ln_3_GaO_6_ has *E_g_*(Ln 4*f*–Ga 4*s*). (c) The third group of Ln_3_GaO_6_ has *E_g_*(O 2*p*–Ln 4*f*). (d) The last group of Ln_3_GaO_6_ has *E_g_*(Ln 4*f* – Ln 4*f*), but they are not found.

We summarize the classification of Ln_3_GaO_6_ in Figure 5. For the first group [Figure 5(a)], Ln_3_GaO_6_ (Ln = La, Nd, Gd, Tb, Dy, Ho, Er, or Lu) have *E_g_*(O 2*p*–Ga 4*s*), which are less affected by the location of the *f*-levels (|Δ*E_g_*| < 0.3 eV). For the second group [Figure 5(b)], Ce_3_GaO_6_ and Pr_3_GaO_6_ have smaller *E_g_*(Ln 4*f*–Ga 4*s*) obtained by the GGA+U/w.*f* method than those obtained by the GGA/w.o.*f* method owing to the occupied *f*-levels over the O 2*p*-LUMO. For the third group [Figure 5(c)], Ln_3_GaO_6_ (Ln = Pm, Sm, Eu, Tm, or Yb) also have smaller *E_g_*(O 2*p*–Ln 4*f*) obtained by the GGA+U/w.*f* method than those obtained by the GGA/w.o.*f* method because of the unoccupied *f*-levels below the Ga 4*s*-LUMO inside *E_g_*. These computational results suggest that the *f*-electrons should be considered as valence electrons for the second and third groups of Ln_3_GaO_6_. For the last group [Figure 5(d)], we do not find any Ln_3_GaO_6_, which has both occupied and unoccupied *f*-levels inside *E_g_*(O 2*p*–Ga 4*s*). The dependence of the location of *f*-levels for Ln_3_GaO_6_ on the type of Ln is generally consistent with that for Ln_2_O_3_, which is confirmed by GW-level calculations [19,20] or experiments [46].

Different features of *f*-levels depending on the type of Ln of Ln_3_GaO_6_ suggest different types of applications. The deep unoccupied *f*-levels of the third group of Ln_3_GaO_6_ (Ln = Pm, Sm, Eu, Tm, or Yb) may have a smaller excitation energy between the HOMO and the unoccupied *f*-level than other Ln_3_GaO_6_. For example, (Gd_1−*x*_Eu*_x_*)_3_GaO_6_ compounds can be used as phosphors [48]. On the other hand, the first group of Ln_3_GaO_6_ (Ln = La, Nd, Gd, Tb, Dy, Ho, Er, or Lu) may have common properties of wide-gap compounds, whose optical absorption, transport properties, and magnetic properties are not affected by the *f*-levels of Ln. Henceforth, we focus on the first group of eight Ln_3_GaO_6_ (Ln = La, Nd, Gd, Tb, Dy, Ho, Er, or Lu) using only the GGA/w.o.*f* calculations, which are much faster than the GGA+U/w.*f* calculations because the latter often suffers from slow convergence due to the localized *f*-levels and spin polarizations.

### 3.3. Energetic and dynamical stability

**Figure 6.**
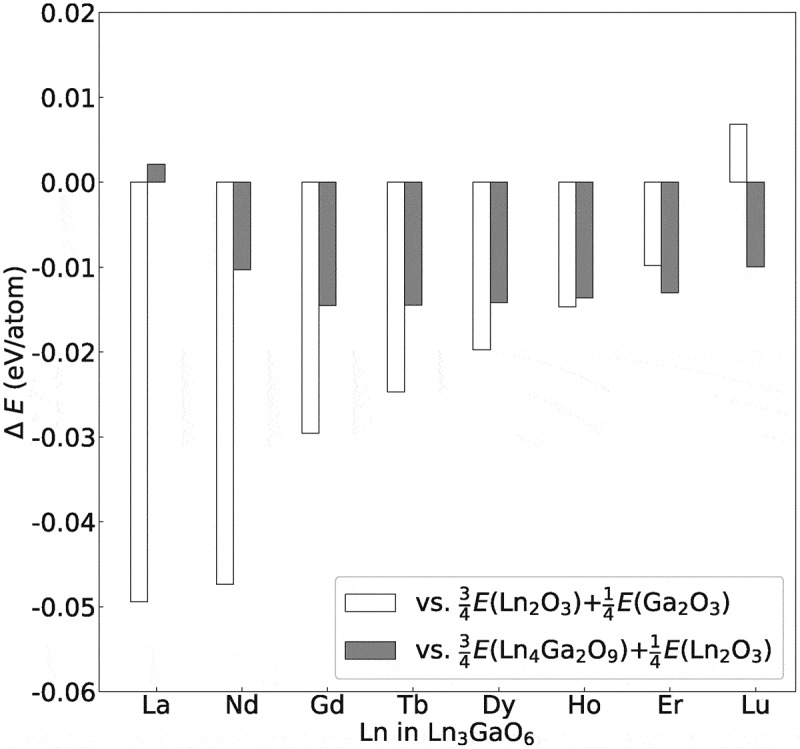
Energy differences (eV/atom) of eight Ln_3_GaO_6_ (Ln = La, Nd, Gd, Tb, Ho, Dy, Er, or Lu) with respect to their competing reference states, namely, the coexisting states of ¾(Ln_2_O_3_) + ¼(Ga_2_O_3_) and ¾(Ln_4_Ga_2_O_9_) + ¼(Ln_2_O_3_).

Figure 6 shows the energies of Ln_3_GaO_6_ (Ln = La, Nd, Gd, Tb, Dy, Ho, Er, or Lu) compared with those of two competing reference states. For the competing reference states, first, the coexisting state of binary sesquioxides of Ln_2_O_3_ and Ga_2_O_3_ (space group *C*2/*m*) is employed. Second, the coexisting state of Ln_4_Ga_2_O_9_ (space group *P*2_1_/*c*) and Ln_2_O_3_ is employed. According to phase diagrams in experimental studies [49–51], the coexisting state of La_4_Ga_2_O_9_ (space group *P*2_1_/*c*) and La_2_O_3_ can be formed at the composition of La_3_GaO_6_.

In the result, seven Ln_3_GaO_6_ except for Lu_3_GaO_6_ are energetically more stable than their competing reference states of Ln_2_O_3_ and Ga_2_O_3_. The absolute energy differences tend to decrease with increasing atomic number of Ln. Lu_3_GaO_6_ is energetically slightly less stable than their corresponding coexisting states of Ln_2_O_3_ and Ga_2_O_3_.

Compared with the coexisting state of Ln_4_Ga_2_O_9_ and La_2_O_3_, the seven Ln_3_GaO_6_ series, except for La_3_GaO_6_, are energetically more stable. The energy of La_3_GaO_6_ is slightly higher (0.002 eV/atom) than that of the coexisting state of La_4_Ga_2_O_9_ and La_2_O_3_. This small energy difference implies a possibility of synthesis of La_3_GaO_6_ as a single phase depending on the synthesis process, such as extrinsic doping and changing of thermodynamic variables.

To summarize, the six Ln_3_GaO_6_ (Ln = Nd, Gd, Tb, Dy, Ho, or Er) are more stable than the two reference states. This computational result on the energetic stability is consistent with the experimental reports. These six oxides on top of Sm_3_GaO_6_ and Eu_3_GaO_6_ were synthesized as single phases by sintering binary sesquioxide powders at 1400 °C [15] or by crystallization of reactive high temperature hydroxide melt [17].

**Figure 7.**
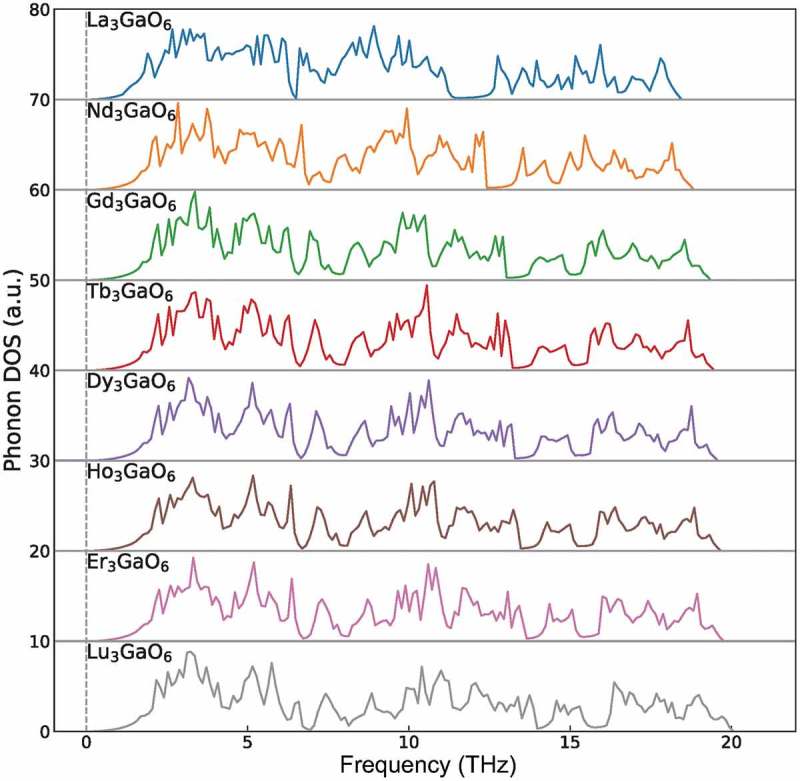
Phonon DOS of eight Ln_3_GaO_6_ (Ln = La, Nd, Gd, Tb, Ho, Dy, Er, or Lu). The vertical dashed line denotes zero phonon frequency. No imaginary phonon frequencies are found, which indicates that these eight oxides are dynamically stable.

We also investigate the dynamical stability of the eight Ln_3_GaO_6_ (Ln = La, Nd, Gd, Tb, Dy, Ho, Er, or Lu) by confirming whether imaginary phonon frequencies on the phonon DOS exist or not. As Figure 7 shows, no imaginary phonon frequencies are found for all the eight Ln_3_GaO_6_, although we confirmed that two of them (La_3_GaO_6_ and Lu_3_GaO_6_) are not energetically in the ground-state. The dynamical stabilities of these oxides suggest that they can also exist as metastable states if they can be once synthesized.

To investigate the stability of these Ln_3_GaO_6_, we consider Pauling’s radius ratio rule [52]. This rule suggests that favorable CNs of cations in the crystal structure depend on the ionic radius ratio (*r*_Ln3+_/*r*_O2−_) of cations (Ln in this study) and anions (O in this study, 1.4 Å [53]). According to this rule, the conditions of 0.41 ≤ *r*_Ln3+_/*r*_O2−_ < 0.59 and 0.59 ≤ *r*_Ln3+_/*r*_O2−_ < 0.73 correspond to favorable CNs of Ln of six and seven, respectively [54]. As shown in Figure 2(b), in general, *r*_Ln3+_ decreases with increasing atomic number of Ln with values of 0.86 (Lu)–1.03 (La) Å. These values can be converted to *r*_Ln3+_/*r*_O2−_ of 0.61 (Lu)–0.74 (La). This suggests that as *r*_Ln3+_ is larger (the atomic number of Ln is smaller), it prefers to form the CN of cation of seven rather than of six. The CNs of Ln of Ln_3_GaO_6_ and Ln_2_O_3_ are seven and six, respectively. Therefore, as *r*_Ln3+_/*r*_O2−_ value is larger (the atomic number of Ln is smaller), Ln_3_GaO_6_ is preferred to be formed rather than binary oxides, which can be confirmed by the energy differences between Ln_3_GaO_6_ and binary oxides as shown in Figure 6. Lu_3_GaO_6_ becomes less stable than the binary oxide because its *r*_Ln3+_/*r*_O2−_ (0.61) is decreased to near the limit of 0.59.

### 3.4. Formation energies of V_O_

Experimental reports [13,14] on Nd_3_GaO_6_ and Gd_3_GaO_6_ doped with acceptor dopants (aliovalent cations with lower valences) such as Sr and Ca (for example, Nd_3−*x*_Sr*_x_*GaO_6−2/*x*_) imply that Ln_3_GaO_6_ have the diffusion mechanism of O based on *V*_O_^2+^. Therefore, it is worthwhile to investigate the possibility of generating *V*_O_^2+^ assuming such type of doping condition with acceptor dopants in Ln_3_GaO_6_. When the negatively charged defects generated by substitution of acceptor dopants for Ln^3+^ or Ga^3+^ are introduced, holes can be increased with shifting the Fermi level from the center of band gap to the HOMO and positively charged defects such as *V*_O_^2+^ (for semiconductors, these defects are sometimes called ‘hole killers’ [55]) can be generated to compensate for the charge imbalance. In this study, we mainly focus on the case where the Fermi level is shifted to HOMO because a few fractions of extrinsic dopants are commonly substituted (high concentration of approximately 10^20^–10^22^/cm^3^) for constituent cations in oxygen-ion conductors [2,13,14].

The *E_v_* values for the eight Ln_3_GaO_6_ (Ln = La, Nd, Gd, Tb, Dy, Ho, Er, or Lu) are summarized in Table 1. We employed *μ*_O_ at the pressure and temperature of 1 atm and 1500 K, respectively, based on the sintering conditions for syntheses of Ln_3_GaO_6_ (1250–1450 °C) [13–15]. The *μ*_O_ value at a certain temperature (*T*) and pressure (*p*) can be obtained as the half energy of O_2_ gas molecule minus 1.75 eV by the following equation:

(5) $$\mu_{\text{O}}\left(T,p\right)=\frac{1}{2}E\left({\text{O}}_{2}\right)+\mu_{\text{O}}\left(T,p{}^{\circ}\right)+\frac{1}{2}kT\text{ln}\left(\frac{p}{p{}^{\circ}}\right)$$

**Table 1.** *E_v_* of eight Ln_3_GaO_6_ (Ln = La, Nd, Gd, Tb, Ho, Dy, Er, or Lu). The computational results in this table are obtained using the GGA/w.o.*f* method. The Fermi level is located at the HOMO (VBM). The values are in eV.

O site		La_3_GaO_6_	Nd_3_GaO_6_	Gd_3_GaO_6_	Tb_3_GaO_6_	Dy_3_GaO_6_	Ho_3_GaO_6_	Er_3_GaO_6_	Lu_3_GaO_6_
	*E_g_*	3.83	3.62	3.59	3.57	3.56	3.54	3.51	3.47
O(*I*)	*E_v_*(*V*_O_^0^)	2.89	2.91	2.92	2.91	2.91	2.91	2.91	2.91
	*E_v_*(*V*_O_^+^)	1.25	1.64	1.50	1.51	1.52	1.53	1.54	1.55
	*E_v_*(*V*_O_^2+^)	−0.49	−0.09	−0.01	0.00	0.01	0.02	0.04	0.04
	*ε*(2+/0)*^a^*	1.69	1.50	1.46	1.46	1.45	1.45	1.44	1.43
O(*II*)	*E_v_*(*V*_O_^0^)	4.92	4.67	4.81	4.83	4.84	4.85	4.87	4.92
	*E_v_*(*V*_O_^+^)	1.65	2.00	1.86	1.89	1.90	1.92	1.94	1.99
	*E_v_*(*V*_O_^2+^)	−0.77	−0.44	−0.40	−0.39	−0.39	−0.39	−0.38	−0.40
	*ε*(1+/0)*^a^*	3.27	2.67	2.95	2.94	2.94	2.93	2.93	2.93
	*ε*(2+/1+)*^a^*	2.42	2.44	2.26	2.28	2.29	2.31	2.32	2.39
O(*III*)	*E_v_*(*V*_O_^0^)	2.68	2.82	2.85	2.86	2.86	2.86	2.86	2.86
	*E_v_*(*V*_O_^+^)	1.07	1.53	1.40	1.41	1.41	1.42	1.43	1.42
	*E_v_*(*V*_O_^2+^)	−0.71	−0.25	−0.18	−0.17	−0.17	−0.17	−0.14	−0.17
	*ε*(2+/0)*^a^*	1.69	1.54	1.52	1.51	1.51	1.51	1.50	1.52
O(*IV*)	*E_v_*(*V*_O_^0^)	2.79	2.86	2.89	2.89	2.89	2.89	2.90	2.90
	*E_v_*(*V*_O_^+^)	1.50	1.90	1.81	1.83	1.84	1.85	1.88	1.90
	*E_v_*(*V*_O_^2+^)	0.55	1.00	1.16	1.20	1.23	1.26	1.31	1.36
	*ε*(1+/0)*^a^*	1.29	0.96	1.08	1.06	1.05	1.04	1.02	1.00
	*ε*(2+/1+)*^a^*	0.95	0.90	0.65	0.63	0.61	0.59	0.57	0.54

*^a^* Locations are counted from the O 2*p*-HOMO.

where *μ*_O_(*T, p*°) can be taken from thermodynamic tables (see the values in other temperatures in Supplementary Table S3) at the standard pressure of 1 atm [56,57]. In addition, the dielectric constants of these eight La_3_GaO_6_, which are used for the image charge correction [34,35], range from 14.6 to 19.4 as summarized in Supplementary Table S4.

**Figure 8.**
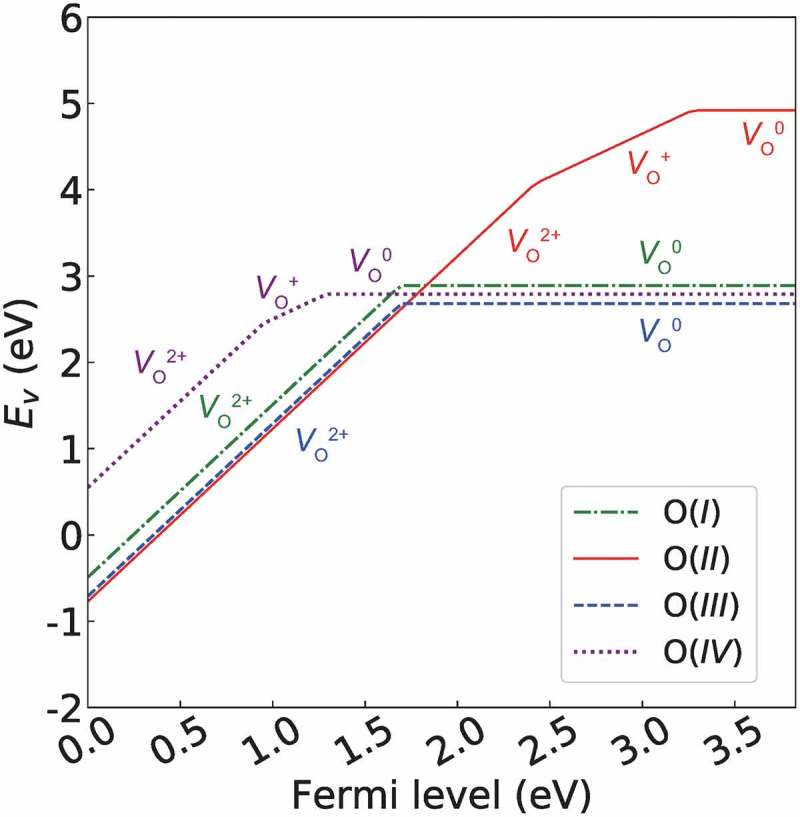
*E_v_* as a function of Fermi level of La_3_GaO_6_. *μ*_O_ is set to be ½*E*(O_2_) − 1.75 eV to describe the pressure and temperature at 1 atm and 1500 K, respectively. The other Ln_3_GaO_6_ (Ln = Nd, Gd, Tb, Dy, Ho, Er, or Lu) series showed a similar trend (see Supplementary Figures S3). The gradient of the lines denotes the charge of the defect.

First, we analyze *E_v_* as a function of the Fermi level of La_3_GaO_6_ as shown in Figure 8. The three types of *E_v_*(*V*_O_^0^) for the O(*I*), O(*III*), and O(*IV*) sites, which have both O–Ln and O–Ga bonds, are all similar at 2.68–2.89 eV, whereas the *E_v_*(*V*_O_^0^) from the O(*II*) site, which has only O–Ln bonds, is much larger at 4.92 eV. When the Fermi level is at the center of *E_g_* or shifted into the LUMO, the *E_v_*(*V*_O_^0^) values for the O(*I*), O(*III*), and O(*IV*) sites are much lower than those of *E_v_*(*V*_O_^+^) and *E_v_*(*V*_O_^2+^). In contrast, when the Fermi level is shifted into the HOMO (assuming that acceptor dopants are introduced to substitute for cations, so that positive charges try to compensate for the charge imbalance), the *E_v_*(*V*_O_^2+^) value for the O(*II*) site is lower than those of the other sites by 0.06–1.04 eV at the same Fermi level, although the other sites also have lower *E_v_*(*V*_O_^2+^) than *E_v_*(*V*_O_^+^) and *E_v_*(*V*_O_^0^) at this region. When the Fermi level is near the HOMO, the *E_v_*(*V*_O_^2+^) values for the three types of O sites except for O(*IV*) site become even negative values, which imply a spontaneous generation of *V*_O_^2+^.

The thermodynamic transition points, which the charge of *V*_O_ changes, *ε*(2+/1+), *ε*(1+/0), and *ε*(2+/0) are all deeply formed. The highest thermodynamic transition point, which is *ε*(1+/0) for the O(*II*) site, forms a deep level of ~0.6 eV below the LUMO.

We compare the *E_v_* values among the eight Ln_3_GaO_6_. The *E_v_* values as a function of the Fermi level for the eight Ln_3_GaO_6_ are also shown in Supplementary Figure S3. The trends in the change of stable charge of *V*_O_ according to the Fermi level are similar to that of La_3_GaO_6_ as abovementioned. When the Fermi level is located at the center of *E_g_*, the most stable is *V*_O_^0^ at the O(*III*) site with *E_v_* values of 2.68–2.86 eV, which are similar to each other for Ln_3_GaO_6_. When the Fermi level is close to the LUMO and HOMO, *V*_O_^0^ and *V*_O_^2+^ are energetically favorable, respectively. The most energetically stable *V*_O_^2+^ is preferred for the O(*II*) site, which has only O–Ln bonds. As shown in Supplementary Figure S4, the top of the HOMO is mainly formed by the O(*II*) site. Therefore, the lower *E_v_* value for the O(*II*) site than those of the other O site is reasonable because the *E_v_* value indicates the energy required for breaking the O–cation bonds and removing O out of the oxide. When the Fermi level is located at the center of *E_g_*, the *E_v_* values of *V*_O_^2+^ at the O(*II*) sites range from 3.06 to 3.19 eV, which are also similar to each other for Ln_3_GaO_6_. When the Fermi level is located at the HOMO, the *E_v_* value of *V*_O_^2+^ at the O(*II*) site of La_3_GaO_6_ ranges from 0.33 to 0.38 eV. This is lower than those of the other seven Ln_3_GaO_6_ because the *E_g_* value of La_3_GaO6 is ~0.2 eV larger so that the decrease in the *E_v_* value with the gradient of −2 is additional in the range of larger *E_g_*. The *E_v_*(*V*_O_^2+^) values for the O(*I*), O(*II*), and O(*III*) sites of Ln_3_GaO_6_ are negative or close to zero, which implies a spontaneous generation of *V*_O_^2+^. This suggests that Ln_3_GaO_6_ can easily generate *V*_O_^2+^ when acceptor dopants substitute for Ln^3+^ or Ga^3+^. In addition, when the pressure of O becomes lower, the *E_v_* value for *V*_O_ further decreases as indicated by Equations (1) and (5). Positive charges such as holes and *V*_O_^2+^ compete with each other to compensate for the limited concentration of negative charged defects. Therefore, the generation of *V*_O_^2+^ can be increased by decreasing the pressure of the oxygen source.

### 3.5. Migration barrier energy (E_m_) of V_O_

There are various migration paths of *V*_O_^2+^ in Ln_3_GaO_6_. Instead of investigating all types of paths for all types of Ln_3_GaO_6_, we chose La_3_GaO_6_ as a representative sample and obtained *E_m_* for all possible migration paths considering the nearest-neighboring O sites. Table 2 presents the *E_m_* values for possible migration paths of La_3_GaO_6_. We named the path after the type of *V*_O_^2+^ site in the initial state for the CI-NEB in the order of O–O distances. The O(*II*) site is energetically the most stable for *V*_O_^2+^, so that this site is considered as a reference. When we consider the *E_m_* value on the migration paths between two O sites other than the O(*II*) site, we added the energy difference (Δ*E_ref_*) between the more stable O site (between initial and final states) on the migration path and the O(*II*) site.

**Table 2.** *E_m_* for various paths of eight La_3_GaO_6_. The computational results in this table are obtained using the GGA/w.o.*f* method. IS, TS, and FS denote the initial, transition, and final states for CI-NEB, respectively.

*V*_O_^2+^ site of IS*^a^*	Path	*V*_O_^2+^ site of FS	E(FS) − E(IS) (eV)	O site distance (Å)	*E_m_* (eV)*^b^*	E(TS) − E(IS) with *V*_O_^2+^[O(*II*)] (eV)*^c^*	Index
O(*I*)	*I*-1	O(*I*)	0.00	2.880	0.62	0.89	
[+0.27 eV]	*I*-2	O(*III*)	−0.16	2.950	0.51	0.63	
	*I*-3	O(*II*)	−0.27	2.987	1.03	1.03	
	*I*-4	O(*IV*)	1.02	3.187	1.24	1.51	
	*I*-5	O(*IV*)	1.02	3.298	1.89	2.16	
	*I*-6	O(*IV*)	1.02	3.304	2.12	2.38	
	*I*-7	O(*I*)	0.00	3.407	1.02	1.29	
	*I*-8	O(*II*)	−0.27	3.462	0.94	0.94	
O(*II*)	*II*-1	O(*II*)	0.00	2.867	0.59	0.59	The lowest *E_m_*
[+0.00 eV]	*II*-2	O(*I*)	0.27	2.987			Same as path *I*-3
	*II*-3	O(*II*)	0.00	3.004	1.02	1.02	
	*II*-4	O(*III*)	0.11	3.269	0.76	0.76	
	*II*-5	O(*I*)	0.27	3.462			Same as path *I*-8
O(*III*)	*III*-1	O(*I*)	0.16	2.950			Same as path *I*-2
[+0.11 eV]	*III*-2	O(*III*)	0.00	3.048	0.74	0.85	
	*III*-3	O(*IV*)	1.18	3.086	1.67	1.78	
	*III*-4	O(*II*)	−0.11	3.269			Same as path *II*-4
	*III*-5	O(*IV*)	1.18	3.271	1.66	1.77	
O(*IV*)	*IV*-1	O(*III*)	−1.18	3.086			Same as *III*-3
[+1.18 eV]	*IV*-2	O(*I*)	−1.02	3.187			Same as *I*-4
	*IV*-3	O(*III*)	−1.18	3.271			Same as path *III*-5
	*IV*-4	O(*I*)	−1.02	3.298			Same as path *I*-5
	*IV*-5	O(*I*)	−1.02	3.304			Same as path *IV*-5

*^a^* Δ*E_ref_*, which can be defined as E(IS) − E(IS) with *V*_O_^2+^[O(*II*)], with a unit of eV are in the brackets.

*^b^* Larger value between *E*(TS) − *E*(IS) and *E*(TS) − *E*(FS).

*^c^* E(IS) with *V*_O_^2+^[O(*II*)] is the most stable among four types of O sites.

We identify five migration paths with the lowest *E_m_* value in La_3_GaO_6_, namely, paths *II*-1, *II*-4, *II*-5 (*I*-8), *III*-1 (*I*-2), and *III*-2, which are between O(*II*)–O(*II*), O(*II*)–O(*III*), O(*II*)–O(*I*), O(*III*)–O(*I*), and O(*III*)–O(*III*) sites, respectively. Then, we also compute the *E_m_* values on the five migration paths for the other seven Ln_3_GaO_6_ (Ln = Nd, Gd, Tb, Dy, Ho, Er, or Lu). These five types of migration paths are displayed in Supplementary Figure S5. Because the O(*IV*) site has Δ*E_ref_* of ~1 eV, which is higher than various *E_m_, V*_O_^2+^ may rarely reach this site.

**Figure 9.**
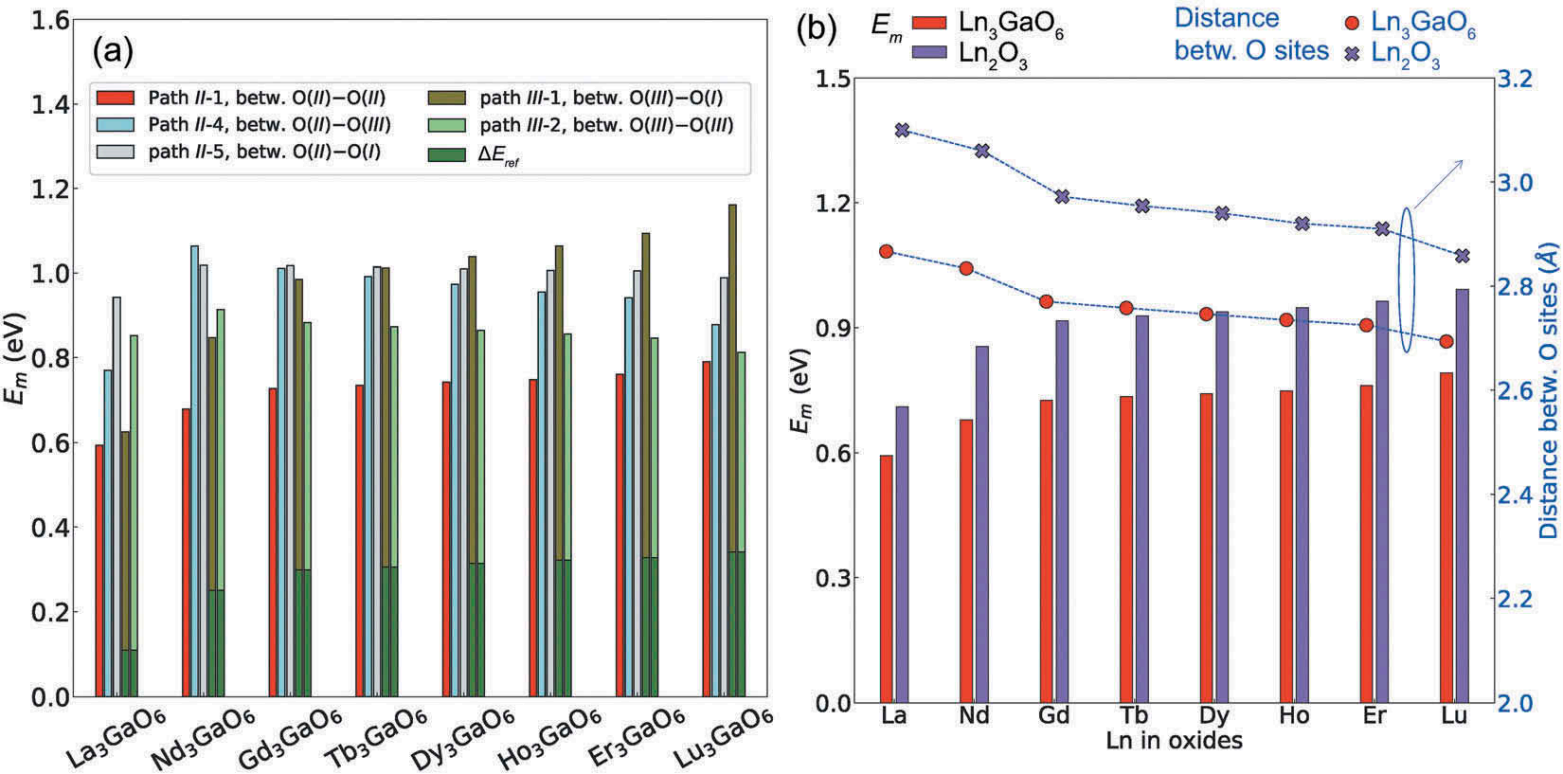
(a) *E_m_* for various migration paths of eight Ln_3_GaO_6_ (Ln = La, Nd, Gd, Tb, Ho, Dy, Er, or Lu) and (b) the lowest *E_m_* of the eight Ln_3_GaO_6_ and their counterpart Ln_2_O_3_ (left vertical axis) and the distances between O sites for the migration path in the crystal structure (right vertical axis).

Figure 9(a) shows the *E_m_* values for the five migration paths of the eight Ln_3_GaO_6_. As abovementioned, we additionally considered the Δ*E_ref_* for the paths *III*-1 and *III*-2 because *V*_O_^2+^ at the O(*III*) or O(*I*) sites are less stable than at the O(*II*) site. Note that those energy differences are slightly larger than those summarized in Table 1 because the image charge correction is not applied for the CI-NEB method.

The lowest *E_m_* value among the five migration paths is found to be path *II*-1, which is between O(*II*)–O(*II*) (see Supplementary Figure S5). In addition, path *II*-1 is delocalized and connected to the neighboring cell; therefore, this can be the dominant migration path. In contrast, paths *II*-4, *II*-5, and *III*-1 with higher (*E_m_*+Δ*E_ref_*) than the *E_m_* value of path *II*-1 are localized and not connected; therefore, complex combinations of paths for migrating *V*_O_^2+^ to reach the neighboring cell are required. Path *III*-2 is delocalized and connected to the neighboring cell; however, this path is formed between the less stable O(*III*) sites. To observe total ionic movements on various migration paths with time scale, other methods such as kinetic Monte Carlo [58,59] and molecular dynamics are necessary. We used FPMD in this study; thus, we will discuss the *D*_O_ that is obtained from the total ionic movements by FPMD in the next subsection.

La_3_GaO_6_ has the lowest *E_m_* value on migration path *II*-1 among the eight Ln_3_GaO_6_. Then, as the atomic number of Ln increases, the *E_m_* values of the eight Ln_3_GaO_6_ also increase. The dependence of *E_m_* on the atomic number of Ln is basically not changed when larger computational cells are used (with a little reduction of less than 0.07 eV) as shown in Supplementary Figure S6. Migration path *II*-1 is neighbored with Ln; therefore, the space for the migration path is related to *r*_Ln3+_. The *E_m_* value on migration path *III*-1 also increases with the increase in the atomic number of Ln. On the other hand, the *E_m_* values on the other three migration paths slightly change or slightly decrease with increasing atomic number of Ln. However, the migration of *V*_O_^2+^ may be dominated by the lowest *E_m_*; therefore, it can be expected that La_3_GaO_6_ might have the most active migrations of *V*_O_^2+^ followed by Nd_3_GaO_6_.

To the best of our knowledge, binary Ln_2_O_3_ compounds are not known to be applicable as oxygen-ion conductors. To determine the effect of the existence of Ga, we compare the distance between O sites neighbored with only Ln as shown in Figure 9(b). As abovementioned, path *II*-1 is between the O(*II*) sites that only have the chemical bonds with only Ln. It is noticeable that the distances between the O(*II*) sites in the eight Ln_3_GaO_6_ are shorter than the minimum distances between the O sites in the counterpart Ln_2_O_3_ by 0.16–0.23 Å (6–8%). In this connection, the *E_m_* values for the migration path between these O sites of the eight Ln_3_GaO_6_ are much lower than those of the counterpart Ln_2_O_3_ by 0.12–0.23 eV (17–21%). Considering the smaller volumes per atom of Ln_3_GaO_6_ than those of Ln_2_O_3_ (Figure 2(b)), we suggest that the advantage of participation of Ga in Ln_3_GaO_6_ is to shrink the migration distance for *V*_O_ and decrease *E_m_*, which may result in more active diffusion of *V*_O_.

### 3.6. Oxygen-ion diffusivity (D_O_) and conductivity (σ_O_)

*D*_O_ was calculated to investigate the total movement of O among the eight Ln_3_GaO_6_ possessing *V*_O_^2+^ by FPMD. The MSDs of O for the computation of *D*_O_ are displayed in Supplementary Figure S7. To accelerate the O diffusion, we chose a high temperature of 1873 K. Here, 1873 K is a little lower temperature than the melting temperature reported in the La_2_O_3_–Ga_2_O_3_ phase diagram [49–51]. We confirm that MSDs of Ln and Ga, which are more than 1 Å^2^, are not found during the FPMD for the eight Ln_3_GaO_6_ (not shown).

To compare the computed *σ*_O_ with the experimental values at a lower temperature of 800 °C, we extrapolated *σ*_O_ using the *D*_O_ value as follows. The temperature dependence of *D*_O_ can be expressed by the Arrhenius equation:

(6) $$\begin{array}{rl} & D_{O}\left(T\right)=D_{O,0}\exp\left(\frac{-E_{a}}{k_{B}T}\right)or\;\\ & log\;D_{O}\left(T\right)=log\;D_{O,0}-\frac{E_{a}}{2.303k_{B}T} \end{array}$$

where *D*_O,0_ is a pre-exponential factor, *k_B_* is the Boltzmann constant, and *E_a_* is an activation energy. Here, for the *E_a_* of equation 6, we employed the lowest *E_m_* value on path *II*-1 obtained by the CI-NEB method, which will be discussed later. The *D*_O_(*T*) at temperatures lower than 1873 K can be obtained by the following relationship:

(7) $$D_{O}\left(T\right)=D_{O}\left(1873K\right)\exp\left[\frac{-E_{a}}{k_{B}}\left(\frac{1}{T}-\frac{1}{1873}\right)\right]$$

The *σ*_O_(*T*) value can be obtained from *D*_O_(*T*) by the Nernst–Einstein equation at the dilute level as follows [60]:

(8) $$\sigma_{O}\left(T\right)=\frac{D_{O}\left(T\right){\left(ze\right)}^{2}c}{k_{B}T}=\frac{D_{O,0}{\left(ze\right)}^{2}c}{k_{B}T}\exp\left(\frac{-E_{a}}{k_{B}T}\right)$$

where *z* is the valence of oxygen, *e* is the unit charge (1.6 × 10^−19^ C), and *c* is the ionic concentration of O in Ln_3_GaO_6_.

**Figure 10.**
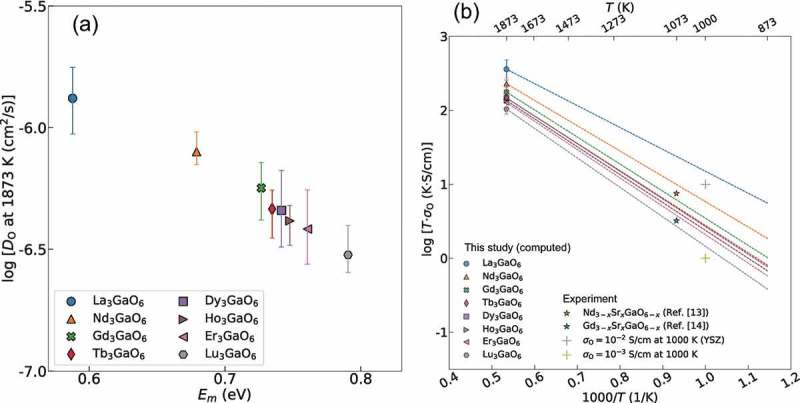
(a) Relationship between *D*_O_ obtained by FPMD at 1873 K and the lowest *E_m_* obtained by CI-NEB, and (b) predicted *σ*_O_ as a function of *T* based on *D*_O_ at 1873 K for eight Ln_3_GaO_6_ (Ln = La, Nd, Gd, Tb, Ho, Dy, Er, or Lu). Solid symbols denote computed or experimentally measured values from references. Dashed lines denote predicted values. The concentration of *V*_O_^2+^ of our computation is ~2.1% from the computational cell of Ln_24_Ga_8_O_47_. The concentration of *V*_O_^2+^ of Nd_2.955_Sr_0.045_GaO_5.9775_ [13] and Gd_2.9_Sr_0.1_GaO_5.95_ [14] are ~0.4 and ~0.8%, respectively. *σ*_O_ of 10^−2^ S/cm at 1000 K is described to show a reference level of YSZ [2]. The *D*_O_ values are averaged from three FPMD trials and error ranges are described between the minimum and maximum values.

The main factors of *D*_O,0_ are the jumping frequency *Г*, which is approximately 10^−12^ Hz in oxides, and the squared distances between the sites α^2^ [61]. We expect that the values of log *D*_O,0_ in Equation (6) for these eight Ln_3_GaO_6_ may be similar to each other considering the same crystal structure and small differences of *α* ^2^ as shown in Figure 9(b). Then, log *D*_O_ at the same temperature may depend on *E_a_*, which is assumed to be the lowest *E_m_*. Figure 10(a) shows the relationship of log *D*_O_ at 1873 K and the lowest *E_m_* (from Figure 9) of Ln_3_GaO_6_. A strong linear correlation between these two properties is found with Pearson’s linear correlation coefficient (*r_p_*) of −0.99 [62]. It is noticeable that La_3_GaO_6_ has the highest *D*_O_ at this temperature, followed by Nd_3_GaO_6_.

Figure 10(b) shows the predicted *σ*_O_ of Ln_3_GaO_6_ as a function of temperature obtained by the above assumption. The experimental *σ*_O_ (0.7 × 10^−2^ S/cm and 0.3 × 10^−2^ S/cm) of Nd_3_GaO_6_ and Gd_3_GaO_6_ (at dry Ar condition) are shown as the highly reported value between various dopants at 1073 K (800 °C), which are obtained at the composition of Nd_2.955_Sr_0.045_GaO_5.9775_ [13] and Gd_2.9_Sr_0.1_GaO_5.95_ [14], respectively. Our calculated *σ*_O_ values of Nd_3_GaO_6_ and Gd_3_GaO_6_ at 800 °C are slightly higher than the experimental values, but lie within the same order of magnitude (approximately 10^−3^–1 × 10^−2^ S/cm).

It is worthwhile to discuss that the differences between our prediction and experimental results might be due to the following reasons. The type and amount of dopants affect the concentrations of *V*_O_^2+^ and *E_a_* for the diffusions of oxygen atoms. We used the concentration of *V*_O_^2+^ of ~2.1% from the computational cell of Ln_24_Ga_8_O_47_, while the concentration of *V*_O_^2+^ of Nd_2.955_Sr_0.045_GaO_5.9775_ [13] and Gd_2.9_Sr_0.1_GaO_5.95_ [14] are ~0.4 and ~0.8%, respectively (assuming that the Sr doping perfectly generates *V*_O_^2+^). In addition, Ref. [14] reported that the Ca-doped Gd_3_GaO_6_ (Gd_3−*x*_Ca*_x_*GaO_6_) shows one order lower *σ*_O_ of 0.04 × 10^−2^ S/cm compared with the counterpart substituted by Sr (Gd_3−*x*_Sr*_x_*GaO_6_), which shows a *σ*_O_ of 0.3 × 10^−2^ S/cm. This may be because *E_a_* can be affected by the type of dopant. Previous studies on doped ceria [63,64] suggest that the atomic radius of the dopant is an important factor for changing *E_a_* and finding the optimum type of dopant. We expect that optimum dopants may be different from each other for Ln_3_GaO_6_ because of different values of *r*_Ln3+_.

The experimental *σ*_O_ also depends on the microstructure of the experimental samples (for example, densification and particle size) and the measuring environment (for example, wet or dry). Ref. [14] reported that a proton conductivity of 1 × 10^−3^ S/cm was also detected for doped Gd_3_GaO_6_ when measured at a slightly lower temperature of 600 °C. When the *σ*_O_ of Gd_2.9_Sr_0.1_GaO_5.95_ was measured at wet Ar condition and 800 °C, *σ*_O_ reportedly increased to 0.9 × 10^−2^ S/cm from 0.3 × 10^−2^ S/cm measured at dry Ar condition. This implies the partial existence of proton conductivity in the total conductivity. We also confirm that protons can be stabilized depending on the environmental variables, such as the type and pressure of source gas, and temperature; therefore, the proton conductivity can be found for other Ln_3_GaO_6_ (see Supplementary Section S.2). When we introduce negatively charged defects by substituting acceptor dopants for Ln^3+^ or Ga^3+^, generation of positively charged defects (proton and *V*_O_^2+^), which may be more dominant than holes considering negative defect formation energies for these defects and large band-gaps of the eight Ln_3_GaO_6_ compounds, compete with each other to compensate for the charge imbalance. Takahashi et al., suggested that the stability of protons and *V*_O_^2+^ is under a trade-off relationship [65]. Therefore, we need to suppress the incorporation of protons to prevent interruption of *V*_O_^2+^ generation for a suitable application of oxygen-ion conductor. As discussed in Supplementary Section S.2, the increase of generation of *V*_O_^2+^ (decrease of *E_v_*) and decrease of incorporation of protons (increase of protonic defect formation energy) can be achieved by increasing temperature or by decreasing oxygen gas and water vapor pressures.

As with the trend of *D*_O_ at 1873 K or the lowest *E_m_* and the atomic number of Ln, the predicted *σ*_O_ at 1000 K tends to decrease with increasing atomic number of Ln. La_3_GaO_6_ and Nd_3_GaO_6_ show larger *σ*_O_ at the same temperature than the other Ln_3_GaO_6_. The predicted *σ*_O_ values of the eight Ln_3_GaO_6_ at 1000 and 1073 K (800 °C) are in the range of 0.1–1.5 × 10^−2^ and 0.3–2.2 × 10^−2^ S/cm, respectively. This computational result suggests a possibility that these eight Ln_3_GaO_6_ can be expected to show a similar order of magnitude of *σ*_O_ in the experiment to a common reference value of ~1 × 10^−2^ S/cm of YSZ at 1000 K [2]. We expect that La_3_GaO_6_ may achieve a higher *σ*_O_ than the experimentally reported Nd_3_GaO_6_ and Gd_3_GaO_6_ if successfully synthesized with the same amount of *V*_O_.

As widely known, *E_a_* is generally obtained using the gradient of Equation (6) with several *D*_O_’s at different temperatures. However, poor statistical data such as insufficient MSD of O are found at temperatures lower than 1873 K. Alternatively, we used the lowest *E_m_* as *E_a_*. In this case, we need to assume that the diffusion mechanism and phase of Ln_3_GaO_6_ are not changed between the computed and target temperatures, and the diffusion of O is mainly found on the suggested migration path with the lowest *E_m_*. We confirm that these eight Ln_3_GaO_6_ are dynamically stable so that *D*_O_ is exclusively obtained for the investigated crystal structures. In addition, we fixed one *V*_O_ in the computational cell; therefore, the diffusion mainly occurs due to *V*_O_ (in fact, an O atom migrates in the opposite direction with *V*_O_). We also confirm that the main diffusion trajectory of O is similar to migration path *II*-1 with the lowest *E_m_* as shown in Supplementary Figures S5 and S8.

Meanwhile, it is worth investigating how the predicted *σ*_O_ at 1000 K changes according to the *E_a_* value because the diffusions of O may not always occur only on the dominant migration path with the lowest *E_m_*. Assuming that the *D*_O_ at 1873 K is fixed because the FPMD already reflects the features of total movements of O, we investigate the change in predicted *σ*_O_ as a function of Δ*E_a_*, which denotes a deviation from the lowest *E_m_* for the *E_a_* of Equations (6)–(8) as shown in Supplementary Figure S9. We confirm that even in the case where Δ*E_a_* is 0.1 eV, most of the predicted *σ*_O_ values at 1000 K for these Ln_3_GaO_6_ keep the same order of magnitude as 10^−3^ S/cm (except for Lu_3_GaO_6_ with *σ*_O_ of 0.8 × 10^−3^ S/cm). It is found that one order of magnitude of *σ*_O_ at 1000 K is decreased when a relatively large value of Δ*E_a_* of ~0.4 eV is increased. Although we exceedingly assume that the diffusions of O mainly occur on the migration path of *V*_O_^2+^ with the highest *E_m_* (0.94–1.06 eV) in Figure 9(a), Δ*E_a_* is less than 0.4 eV. In an experiment, Ref. [13] reported that doped Nd_3_GaO_6_, namely, Nd_2.91_Ca_0.09_GaO_5.955_ and Nd_2.955_Sr_0.045_GaO_5.9775_ showed an *E_a_* value of 0.97 and 1.06 eV, respectively. Ref. [14] reported that doped Gd_3_GaO_6_, namely, Gd_2.9_Ca_0.1_GaO_5.95_ and Gd_2.9_Sr_0.1_GaO_5.95_ showed an *E_a_* value of 0.98 and 0.92 eV at dry Ar condition, respectively. Although there are some variables such as different concentrations of *V*_O_^2+^ and existence of dopants, the differences between the lowest *E_m_* and the *E_a_* of the more optimum reported value in the experiment of Nd_3_GaO_6_ and Gd_3_GaO_6_ are approximately less than ~0.3 eV. Therefore, we expect that the prediction error of *σ*_O_ at 1000 K of our model may be less than one order of magnitude.

Before concluding, we suggest another merit of the investigation of Ln_3_GaO_6_. Recently, there have been many machine learning studies on exploring good functional materials [66–68]. Despite the efficient search by this type of study, one shortcoming is the insufficient number of training data that exclusively have the target variable in a wide range. Although our predicted *σ*_O_ values of Ln_3_GaO_6_ are not the highest among all the reported values, the moderate value of functional property is also useful for an accurate prediction model in a wider search space [68]. We expect that the reported properties of Ln_3_GaO_6_ in this study and other relevant future works in experiments may be able to contribute to the construction of abundant training data for the machine learning models in the search for oxygen-ion conductors.

## 4. Conclusion

We systematically investigated the structural and electronic structure of 15 Ln_3_GaO_6_ (space group *Cmc*2_1_). In general, Ln_3_GaO_6_ have decreasing tendency of the Ln–O (or O–Ln) bond lengths and volumes with increasing the atomic number of Ln and decreasing *r*_Ln3+_. Then, we distinguished eight Ln_3_GaO_6_ (Ln = La, Nd, Gd, Tb, Ho, Dy, Er, or Lu) that have *E_g_*(O 2*p*–Ga 4*s*) from the others that have the localized *f*-levels inside *E_g_*(O 2*p*–Ga 4*s*) with crosschecks on the previous studies on Ln_2_O_3_. These eight Ln_3_GaO_6_ are dynamically stable. Among them, six Ln_3_GaO_6_ (Ln = Nd, Gd, Tb, Ho, Dy, or Er) are also energetically stable than the competing reference states, which is consistent with experimental reports on successful syntheses of a single phase. La_3_GaO_6_ and Lu_3_GaO_6_ are energetically slightly less stable than their competing reference states; however, they might be stabilized within a certain process window considering dynamical stabilities and small energy differences from their competing reference states.

The properties related to *V*_O_ were also analyzed. We confirmed that Ln_3_GaO_6_ may easily generate *V*_O_^2+^ by an extrinsic doping with acceptor dopants considering their low *E_v_* when the Fermi level is near the HOMO region. The CI-NEB results showed that the lowest *E_m_* values of the eight Ln_3_GaO_6_ compounds among various migration paths of *V*_O_^2+^ ranged from 0.59 to 0.79 eV. The *E_m_* values of these Ln_3_GaO_6_ compounds are much lower than the counterpart Ln_2_O_3_, which may be ascribed to the shortened distances between O sites by the participation of Ga^3+^, which has smaller ionic radius. Among the Ln_3_GaO_6_ compounds, La_3_GaO_6_ has the lowest *E_m_*, followed by Nd_3_GaO_6_. The FPMD results showed a strong correlation of the lowest *E_m_* obtained by CI-NEB and the *D*_O_ at 1873 K of Ln_3_GaO_6_. Finally, we predicted the *σ*_O_ at 1073 K (800 °C) based on the computed *D*_O_ and the *E_m_* obtained by CI-NEB and confirmed that the predicted *σ*_O_ and the previously reported experimental values have a similar order of magnitude. In particular, La_3_GaO_6_ shows a higher predicted *σ*_O_ of 2.2 × 10^−2^ S/cm at 1073 K (800 °C) than those of previously reported Nd_3_GaO_6_ and Gd_3_GaO_6_. In addition, these eight Ln_3_GaO_6_ compounds are expected to have a similar *σ*_O_ value as that of the common reference value of 1 × 10^−2^ S/cm of YSZ at 1000 K. Considering that the optimum conditions for YSZ, such as the concentration of dopant, and the processing condition have been developed for decades, we expect that these oxides also have potential as suitable oxygen-ion conductors.

The findings of this study can provide insights into the investigation on Ln_3_GaO_6_-based oxygen-ion conductors. The *σ*_O_ values of these oxides can be further improved by additional cation doping and mixing. In addition, Ln_3_GaO_6_ compounds have lower melting temperatures than YSZ (~1700 °C [49–51,69] vs. >2700 °C [70]), which can provide an advantage of processing at lower temperatures.
