# Supplementary material for: First-principles prediction of high oxygen-ion conductivity in trilanthanide gallates Ln3GaO6
Source: Sci Technol Adv Mater. 2019 Feb 6;20(1):144–59. doi: 10.1080/14686996.2019.1578183 (PMC6407603; doi:10.1080/14686996.2019.1578183)
Supplement: Supplemental Material [file TSTA_A_1578183_SM4002.pdf]

Supplementary Material

First-principles prediction of high oxygen-ion conductivity in trilanthanide gallates Ln_3GaO_6

Joohwi Lee, Nobuko Ohba and Ryoji Asahi

Toyota Central R&D Laboratories, Inc., Nagakute, Aichi 480-1192, Japan

S. 1. Various supplementary figures and tables .... page. 2

S. 2. Stability of protonic defect .... page. 15

S. 1. Various supplementary figures and tables

Table S1. Mean bond lengths of Ln_3GaO_6. Computed results are obtained in this study. Parentheses denote the standard deviation of the bond lengths.

	Method	Bond lengths (Å)							
Ln in Ln_3GaO_6		La	Ce	Pr	Nd	Pm	Sm	Eu	Gd
Ln(*I*)–O [CN = 7]	GGA+U/w.*f*	2.552 (0.161)	2.525 (0.159)	2.500 (0.153)	2.486 (0.154)	2.468 (0.151)	2.449 (0.149)	2.440 (0.146)	2.414 (0.148)
	GGA/w.o.*f*	2.532 (0.162)	2.531 (0.157)	2.504 (0.154)	2.480 (0.152)	2.457 (0.150)	2.441 (0.149)	2.421 (0.147)	2.403 (0.146)
	Exp.[a]				2.466 (0.179)		2.434 (0.167)	2.413 (0.163)	2.410 (0.160)
Ln(*II*)–O [CN = 7]	GGA+U/w.*f*	2.524 (0.102)	2.496 (0.095)	2.475 (0.091)	2.460 (0.087)	2.442 (0.088)	2.425 (0.086)	2.414 (0.074)	2.390 (0.084)
	GGA/w.o.*f*	2.502 (0.098)	2.504 (0.102)	2.477 (0.098)	2.455 (0.095)	2.432 (0.092)	2.416 (0.090)	2.397 (0.087)	2.379 (0.084)
	Exp.[a]				2.444 (0.070)		2.414 (0.075)	2.407 (0.075)	2.386 (0.083)
Ga–O or O–Ga [CN = 4]	GGA+U/w.*f*	1.895 (0.014)	1.892 (0.015)	1.891 (0.015)	1.890 (0.015)	1.889 (0.015)	1.887 (0.017)	1.886 (0.017)	1.885 (0.016)
	GGA/w.o.*f*	1.892 (0.015)	1.894 (0.015)	1.892 (0.015)	1.890 (0.015)	1.889 (0.015)	1.888 (0.016)	1.886 (0.016)	1.885 (0.016)
	Exp.[a]				1.853 (0.090)		1.823 (0.045)	1.853 (0.078)	1.825 (0.033)
O–Ln [CN = 4][b]	GGA+U/w.*f*	2.504 (0.093)	2.476 (0.088)	2.455 (0.088)	2.439 (0.085)	2.421 (0.083)	2.404 (0.082)	2.394 (0.076)	2.368 (0.081)
	GGA/w.o.*f*	2.482 (0.092)	2.484 (0.091)	2.457 (0.089)	2.434 (0.087)	2.412 (0.085)	2.396 (0.083)	2.376 (0.081)	2.358 (0.080)
O–Ln [CN = 5][c]	GGA+U/w.*f*	2.765 (0.007)	2.735 (0.023)	2.700 (0.003)	2.688 (0.005)	2.667 (0.008)	2.647 (0.012)	2.634 (0.001)	2.608 (0.024)
	GGA/w.o.*f*	2.746 (<0.001)	2.738 (0.024)	2.706 (0.027)	2.680 (0.029)	2.654 (0.029)	2.636 (0.030)	2.614 (0.030)	2.594 (0.031)
Ln in Ln_3GaO_6		Tb	Dy	Ho	Er	Tm	Yb	Lu	
Ln(*I*)–O [CN = 7]	GGA+U/w.*f*	2.389 (0.146)	2.368 (0.145)	2.340 (0.145)	2.356 (0.142)	2.332 (0.141)	2.321 (0.139)	2.291 (0.142)	
	GGA/w.o.*f*	2.388 (0.144)	2.374 (0.143)	2.360 (0.142)	2.347 (0.141)	2.334 (0.139)	2.322 (0.139)	2.309 (0.138)	
	Exp.[a]	2.393 (0.161)	2.376 (0.156)	2.363 (0.144)	2.344 (0.159)				
Ln(*II*)–O [CN = 7]	GGA+U/w.*f*	2.365 (0.082)	2.346 (0.079)	2.321 (0.078)	2.334 (0.076)	2.310 (0.076)	2.299 (0.065)	2.270 (0.073)	
	GGA/w.o.*f*	2.365 (0.083)	2.351 (0.081)	2.337 (0.098)	2.325 (0.076)	2.312 (0.075)	2.300 (0.074)	2.288 (0.072)	
	Exp.[a]	2.370 (0.069)	2.361 (0.086)	2.351 (0.081)	2.327 (0.115)				
Ga–O or O–Ga [CN = 4]	GGA+U/w.*f*	1.883 (0.016)	1.882 (0.017)	1.879 (0.018)	1.881 (0.017)	1.879 (0.018)	1.878 (0.019)	1.876 (0.018)	
	GGA/w.o.*f*	1.884 (0.017)	1.883 (0.017)	1.882 (0.017)	1.881 (0.017)	1.880 (0.018)	1.879 (0.018)	1.878 (0.018)	
	Exp.[a]	1.833 (0.069)	1.845 (0.064)	1.833 (0.085)	1.855 (0.077)				
O–Ln [CN = 4][b]	GGA+U/w.*f*	2.344 (0.080)	2.324 (0.080)	2.298 (0.082)	2.312 (0.077)	2.288 (0.076)	2.276 (0.070)	2.247 (0.077)	
	GGA/w.o.*f*	2.343 (0.079)	2.329 (0.078)	2.316 (0.077)	2.304 (0.076)	2.290 (0.075)	2.278 (0.075)	2.266 (0.074)	
O–Ln [CN = 5][c]	GGA+U/w.*f*	2.580 (0.023)	2.556 (0.011)	2.527 (0.004)	2.542 (0.011)	2.515 (0.004)	2.504 (0.002)	2.474 (0.006)	
	GGA/w.o.*f*	2.577 (0.028)	2.561 (0.026)	2.546 (0.021)	2.531 (0.021)	2.516 (0.017)	2.504 (0.014)	2.489 (0.009)	

[a] From [1].

[b] CN = 4 is counted with three Ln and one Ga.

[c] CN = 5 is counted with four Ln and one Ga.

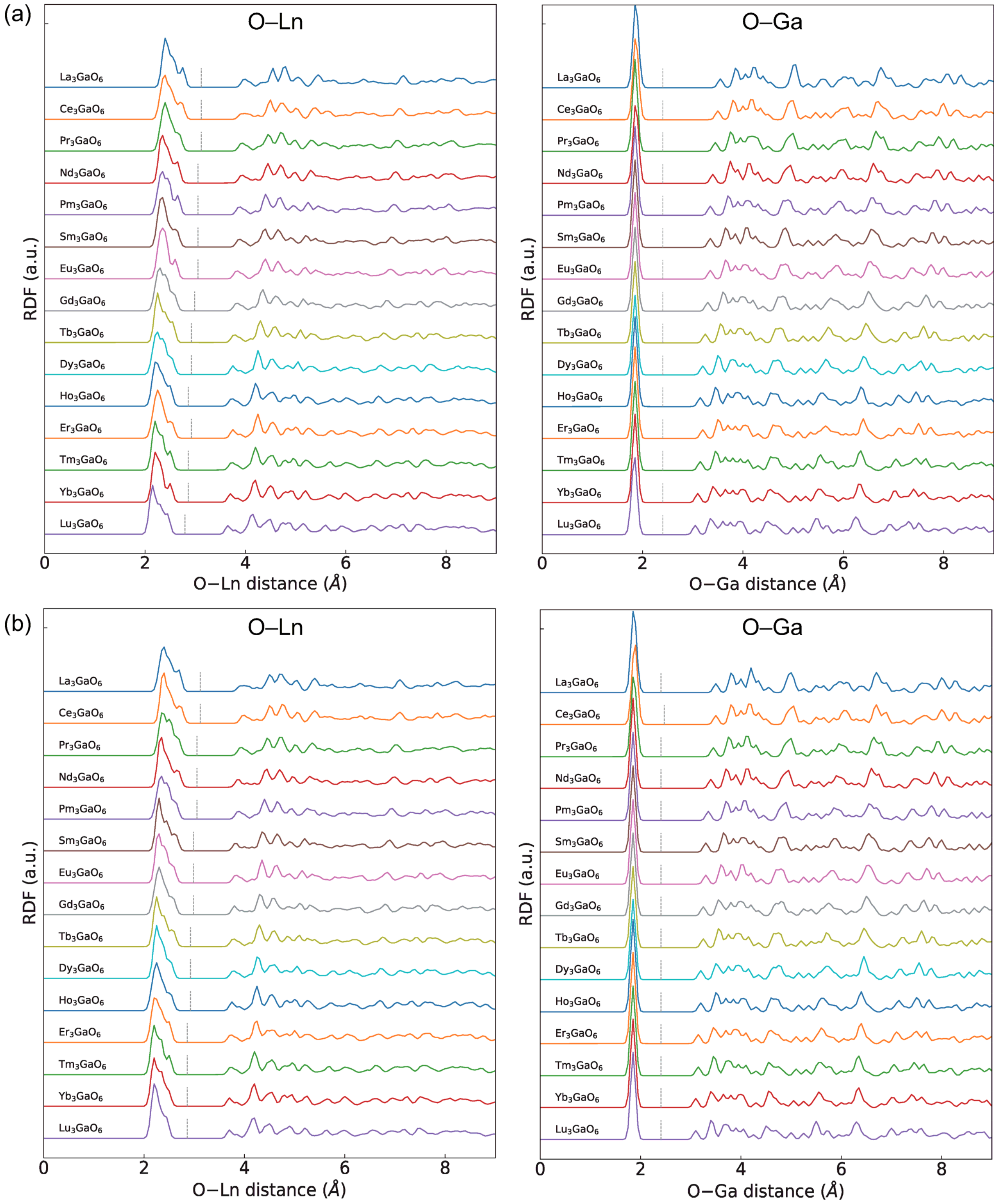

Figure S1. Radial distribution function (RDF) profiles of 15 Ln_3GaO_6 obtained by (a) the GGA+U/w.*f* and (b) GGA/w.o.*f* methods, respectively. The horizontal gray dashed lines indicate the cutoff radius of 1.3 × the maximum peak, which is used for identifying the bond lengths.

Table S2. Lattice parameters of Ln_3GaO_6 in the orthorhombic (conventional) unit-cells and radius of Ln^{3+}. Computed results are obtained in this study.

Ln in Ln_3GaO_6	Method	La	Ce	Pr	Nd	Pm	Sm	Eu	Gd
a (Å)	GGA+U/w.*f*	9.525	9.428	9.348	9.303	9.245	9.173	9.133	9.042
	GGA/w.o.*f*	9.448	9.451	9.356	9.275	9.194	9.137	9.070	9.005
	Exp.[a]				9.182		9.074	9.028	8.991
b (Å)	GGA+U/w.*f*	11.960	11.827	11.724	11.658	11.587	11.500	11.485	11.344
	GGA/w.o.*f*	11.887	11.849	11.730	11.628	11.528	11.456	11.370	11.291
	Exp.[a]				11.540		11.407	11.342	11.280
c (Å)	GGA+U/w.*f*	5.751	5.705	5.667	5.636	5.598	5.575	5.550	5.513
	GGA/w.o.*f*	5.706	5.722	5.676	5.634	5.594	5.565	5.530	5.497
	Exp.[a]				5.575		5.518	5.495	5.480
V ($Å^3$/atom)	GGA+U/w.*f*	16.377	15.903	15.527	15.281	14.992	14.702	14.554	14.138
	GGA/w.o.*f*	16.020	16.020	15.571	15.191	14.822	14.563	14.257	13.974
	Exp.[a]				14.767		14.280	14.068	13.894
Radius of Ln^{3+} (Å)[b]		1.03	1.02	0.99	0.983	0.97	0.958	0.947	0.938

Ln in Ln_3GaO_6	Method	Tb	Dy	Ho	Er	Tm	Yb	Lu
a (Å)	GGA+U/w.*f*	8.954	8.879	8.787	8.843	8.750	8.717	8.608
	GGA/w.o.*f*	8.952	8.902	8.855	8.812	8.763	8.723	8.679
	Exp.[a]	8.934	8.888	8.845	8.801			
b (Å)	GGA+U/w.*f*	11.237	11.153	11.049	11.096	11.002	10.955	10.816
	GGA/w.o.*f*	11.225	11.163	11.105	11.049	10.991	10.940	10.887
	Exp.[a]	11.215	11.151	11.093	11.035			
c (Å)	GGA+U/w.*f*	5.469	5.429	5.382	5.409	5.361	5.339	5.288
	GGA/w.o.*f*	5.470	5.444	5.419	5.396	5.371	5.349	5.326
	Exp.[a]	5.445	5.422	5.403	5.380			
V ($Å^3$/atom)	GGA+U/w.*f*	13.756	13.440	13.065	13.269	12.902	12.747	12.307
	GGA/w.o.*f*	13.742	13.525	13.321	13.134	12.932	12.761	12.579
	Exp.[a]	13.639	13.433	13.253	13.062			
Radius of Ln^{3+} (Å)[b]		0.923	0.912	0.901	0.89	0.88	0.868	0.861

[a] From [1].

[b] From [2].

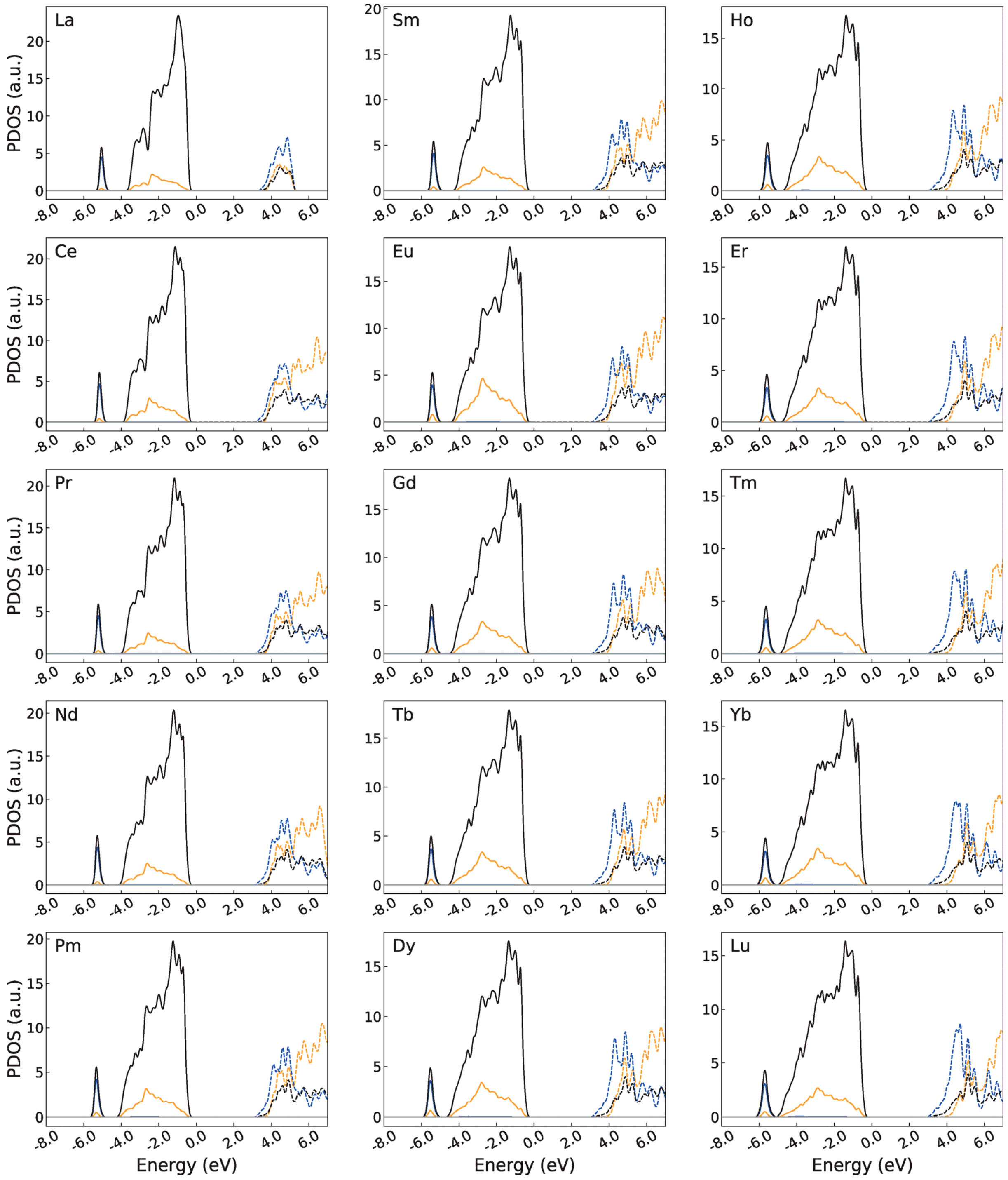

Figure S2. Electronic PDOS of 15 Ln_3GaO_6 obtained by the GGA/w.o.*f* method. To avoid the complexity, only Ln 5*d* (yellow), Ga 4*s* (blue), O 2*p* (black) states are drawn. Energy is shifted by the computational fermi level. Occupied and unoccupied states are drawn as solid and dashed lines, respectively. For easier viewing, the unoccupied Ga 4*s* and O 2*p* states are 15 and 2 times magnified, respectively.

Table S3. Dependence of $\mu_O(T, p^\circ)$ on temperature [3].

Temperature (K)	$\mu_O(T, p^\circ)$ (eV)	Temperature (K)	$\mu_O(T, p^\circ)$ (eV)
0	0.00	900	−0.97
100	−0.07	1000	−1.10
200	−0.17	1100	−1.23
250	−0.22	1200	−1.36
298.15	−0.27	1300	−1.49
300	−0.27	1400	−1.62
350	−0.33	1500	−1.75
400	−0.38	1600	−1.88
450	−0.44	1700	−2.02
500	−0.50	1800	−2.16
600	−0.61	1900	−2.29
700	−0.73	2000	−2.43
800	−0.85		

Table S4. Dielectric constant of eight Ln_3GaO_6 (Ln = La, Nd, Gd, Tb, Ho, Dy, Er, or Lu). The computational results in this table are obtained using the GGA/w.o.*f*. Because the values for three dimensions are similar each other, the dielectric constant is obtained by a geometric mean.

	La_3GaO_6	Nd_3GaO_6	Gd_3GaO_6	Tb_3GaO_6	Dy_3GaO_6	Ho_3GaO_6	Er_3GaO_6	Lu_3GaO_6
$\varepsilon_{r,xx}$	20.9	16.7	15.7	15.5	15.4	15.2	15.0	14.8
$\varepsilon_{r,yy}$	19.3	15.8	15.0	14.9	14.8	14.7	14.4	14.3
$\varepsilon_{r,zz}$	18.0	15.5	15.1	15.0	15.0	14.9	14.8	14.8
$(\varepsilon_{r,xx}\varepsilon_{r,yy}\varepsilon_{r,zz})^{1/3}$	19.4	16.0	15.2	15.1	15.0	14.9	14.7	14.6

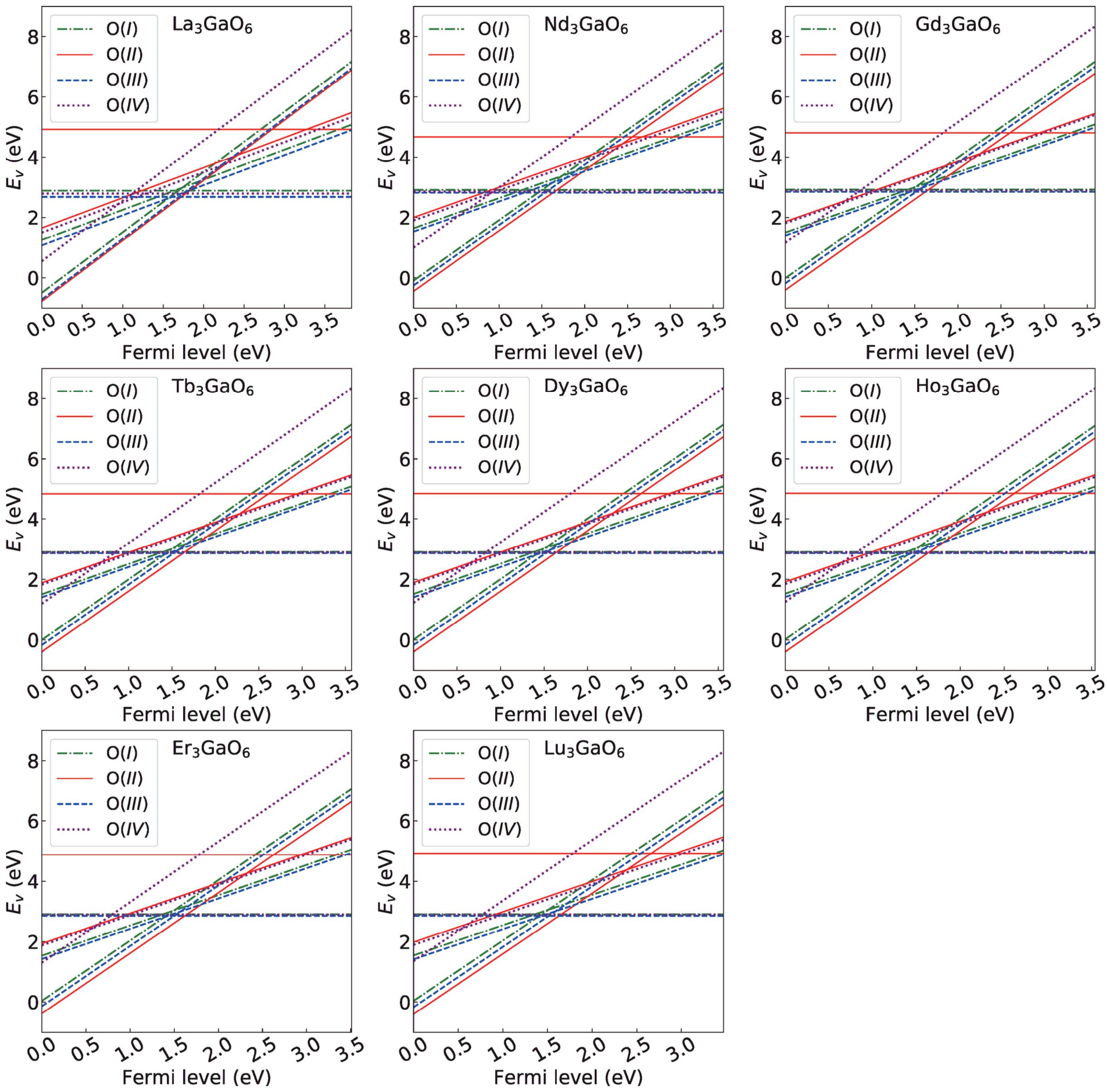

Figure S3. E_v as a function of Fermi level of eight Ln_3GaO_6 (Ln = La, Nd, Gd, Tb, Ho, Dy, Er, or Lu). The μ_O is set to be ½$E(O_2)$ − 1.75 eV to describe the pressure and temperature at 1 atm and 1500 K, respectively. The gradient of the lines denotes the charge of the defect.

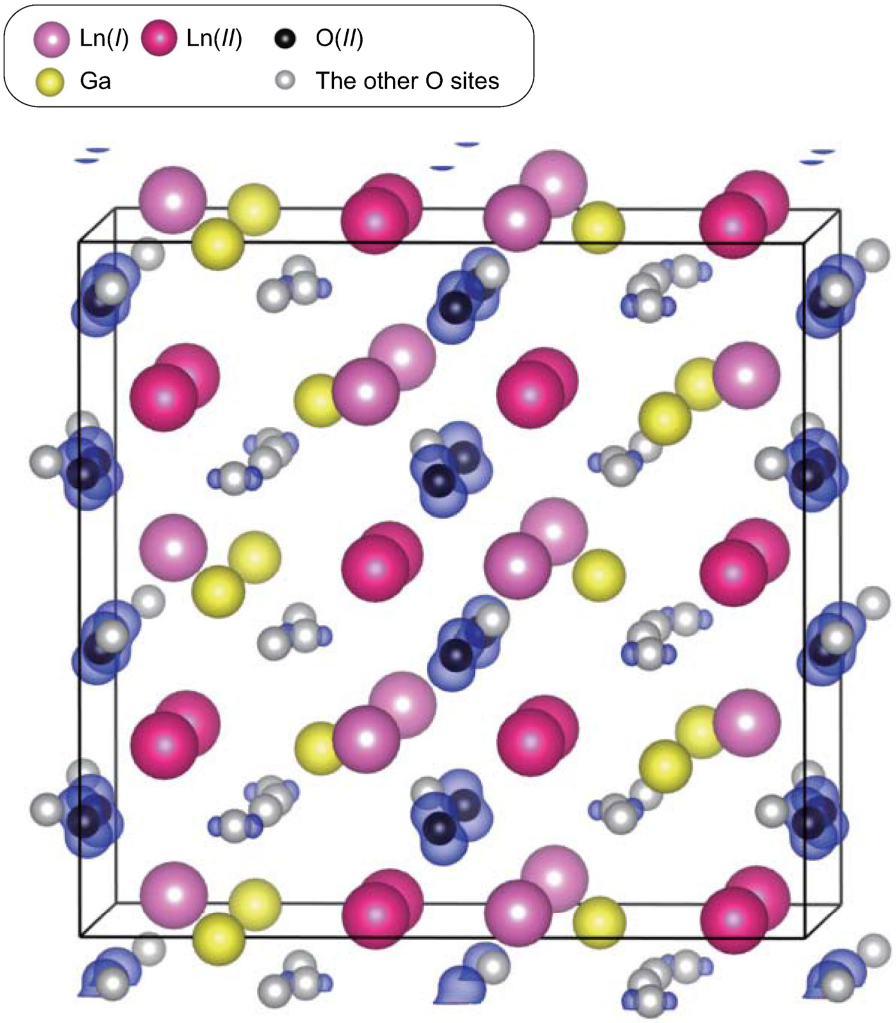

Figure S4. $|\psi|^2$ plots of the top of the HOMO (one band at the VBM). The isosurface level (blue) is 0.005 electrons per $Å^3$. Black and silver colors are used for presenting the O(*II*) and the other O sites to emphasize their contrast, respectively. Atomic configurations and colors of cations are the same as Figure 1. The top of the HOMO is mainly formed by O 2*p* at the O(*II*) site. Similar features are found in the other Ln_3GaO_6 (Ln = Nd, Gd, Tb, Ho, Dy, Er, or Lu).

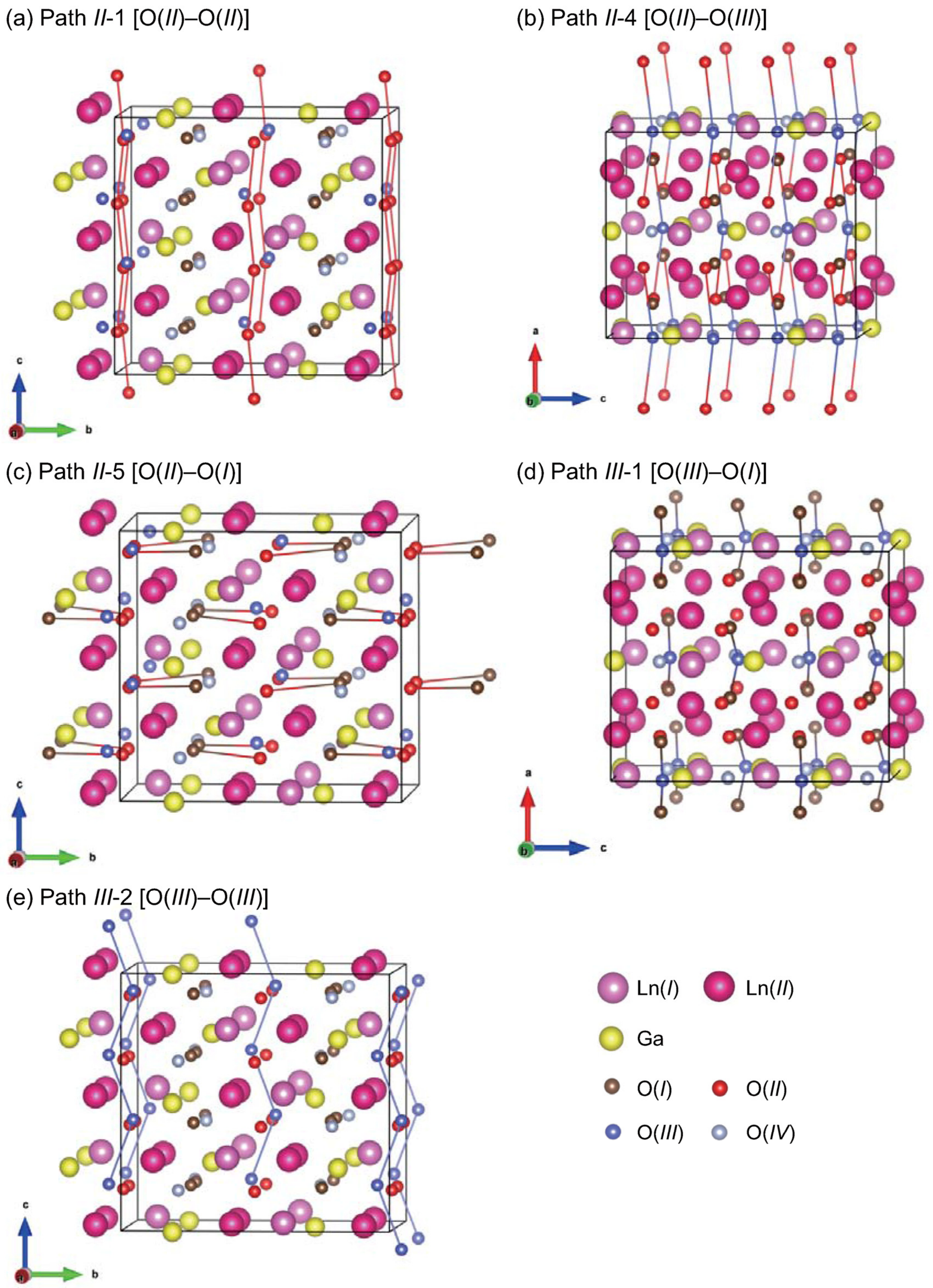

Figure S5. Linear connections between O sites for the migration paths of V_O^{2+}, (a) path *II*-1, (b) path *II*-4, (c) path *II*-5, (d) path *III*-1, and (e) path *III*-2 of Ln_3GaO_6. The path *II*-1 and path *III*-2 are connected to the neighboring cell.

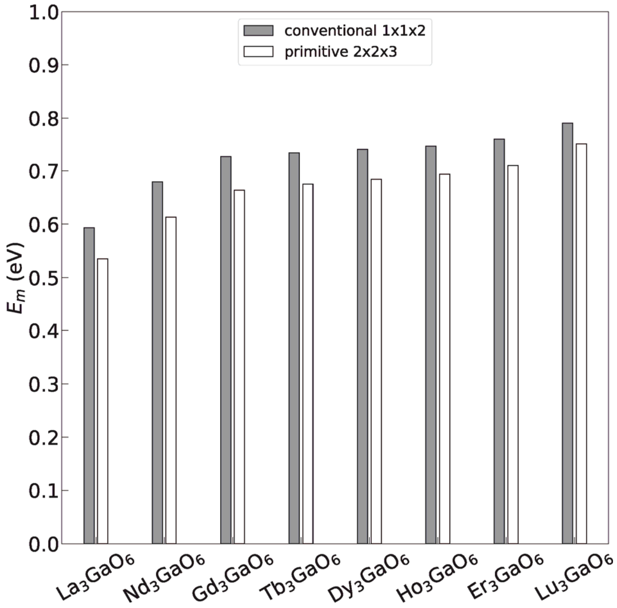

Figure S6. Comparison of the lowest E_m using 1×1×2 convention cell (80 atoms for perfect crystal) and 2×2×3 primitive cell (240 atoms for perfect crystal). The dependence of the E_m on the atomic number of Ln is not changed when larger computational cells are used (with little decrease of less than 0.07 eV).

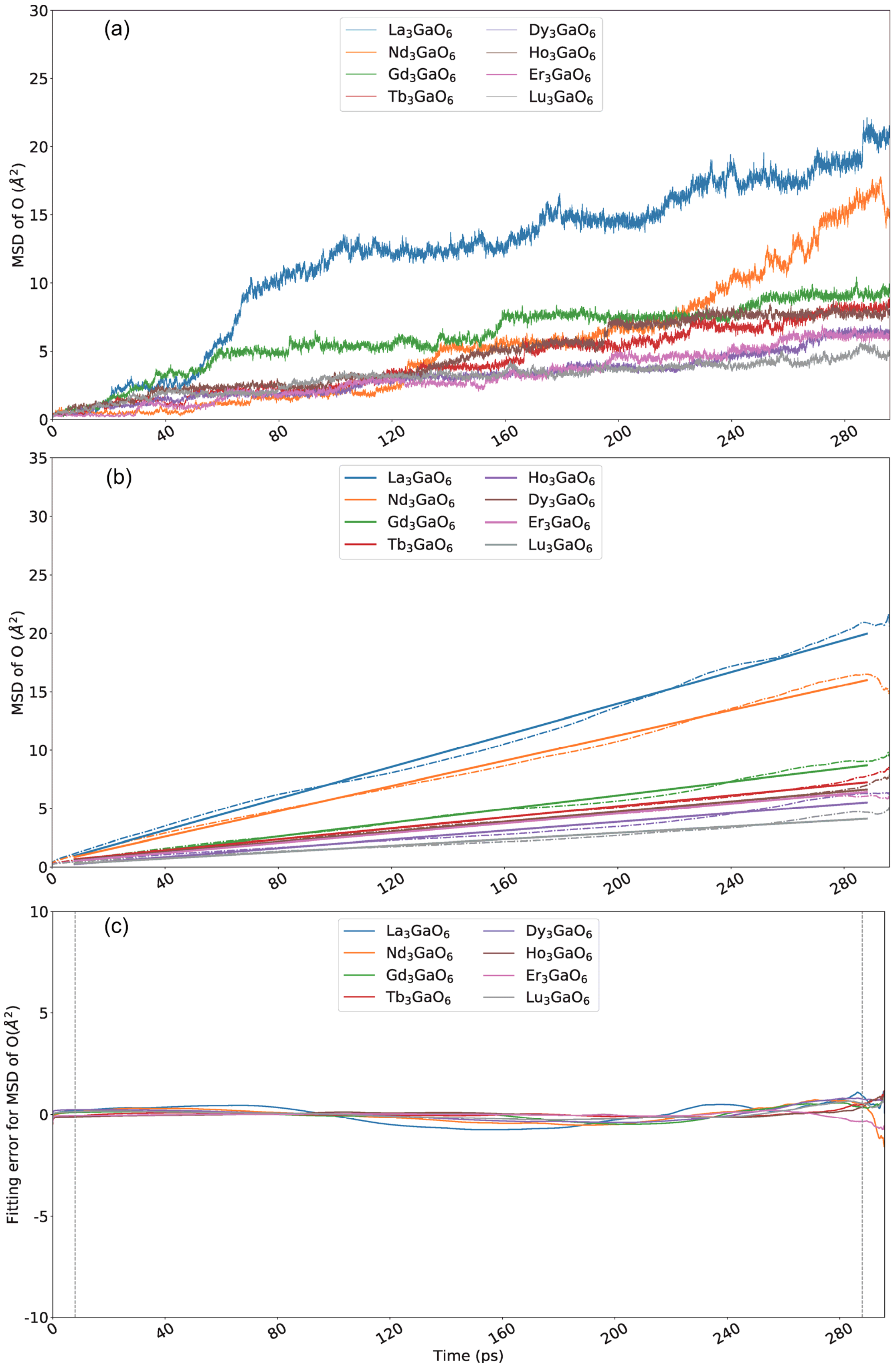

Figure S7. MSD of O as a function of time (*t*) of eight Ln_3GaO_6 (Ln = La, Nd, Gd, Tb, Ho, Dy, Er, or Lu) obtained by FPMD at 1873 K. (a) MSD are collected by using equation 3. (b) MSD is collected by using equation 4. Dashed dot and solid lines denote MSD of O and its linear fitting line, respectively. (c) Fitting error of MSD of O. Dashed vertical gray lines denote sampling range for the linear fitting. The fitting errors are confirmed to be ignorable. One of three trials of the FPMD is viewed.

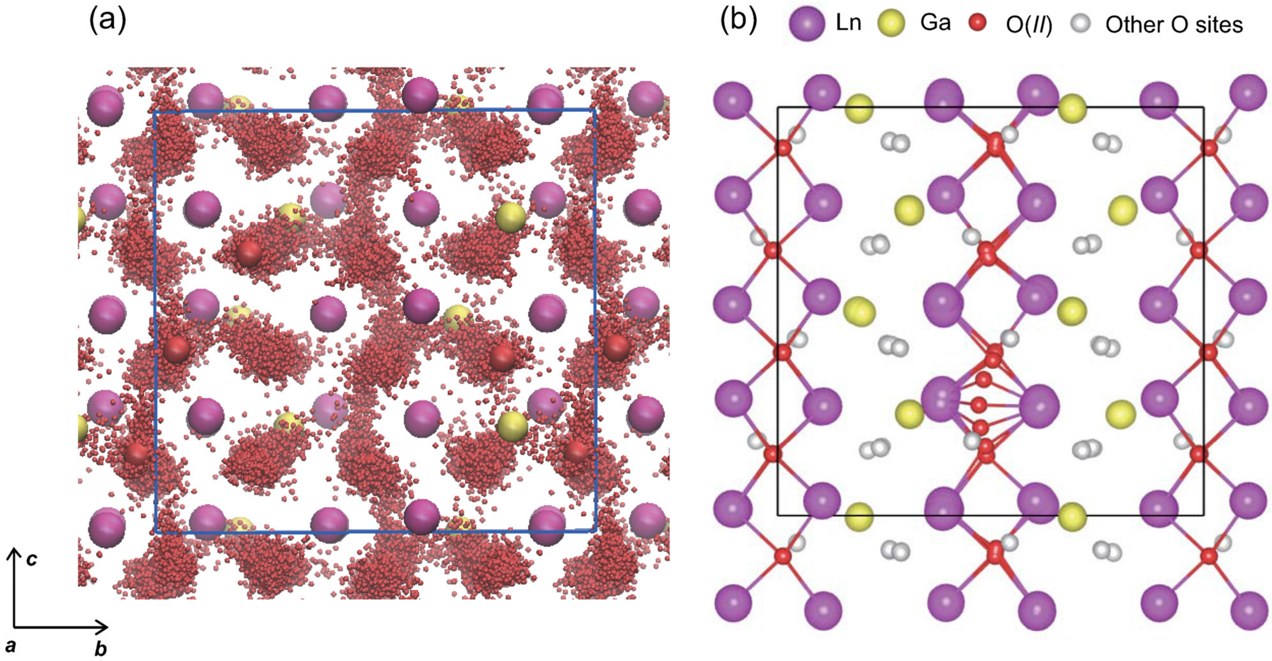

Figure S8. (a) Trajectory of O (small red spheres) of La_3GaO_6 obtained by FPMD at 1873 K with step of 50 cycles (0.1 ps) between 50000–100000 cycles (100–200 ps). La, Ga, and O atoms with the initial positions are colored as pink, yellow, and red, respectively. (b) Trajectory of migration path *II*-1 obtained by CI-NEB, which is also displayed in Supplementary Figure S5. Rectangular boxes denote the computational cells.

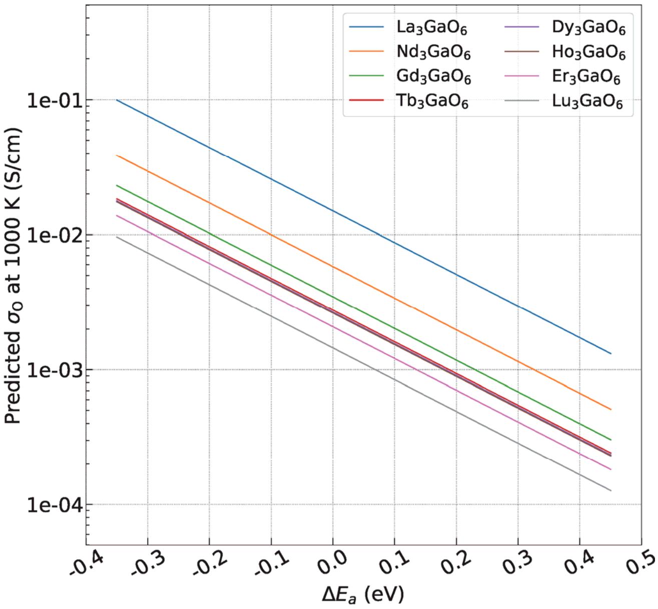

Figure S9. Predicted σ_O at 1000 K in a function of ΔE_a. Here, ΔE_a is for the E_a = the lowest $E_m + \Delta E_a$ in equations 6–8. Note that we fixed D_O at 1873 K in equations 6–8 for this figure because the FPMD already reflect the features of total movements of O.

S. 2. Stability of protonic defect

We considered the stability of proton (H^+) in Ln_3GaO_6 considering the existence of proton conductivity found in the doped Gd_3GaO_6 [4]. As initial locations of H interstitials (H_i), we chose all possible centers of O–cation bonds and found the most stable site using La_3GaO_6. As shown in Supplementary Figure S10, the stable H_i are optimized in different sites from the centers of O–cation bonds, which were set as the initial locations. Stable sites for H^+ (H is attached to an oxygen) and H^- (H is attached to a cation) are different from each other like in other compounds such as SiO_2 [5]. Then, the information of stable sites was applied to the calculations for the other Ln_3GaO_6. Note that there can be other initial locations of H_i sites because small hydrogen atoms can be incorporated in many available sites such as voids and anti-bonding sites [6].

The defect formation energy of H_i (E_H) can be obtained by the following equation:

$$E_{\mathrm{H}} = E(\mathrm{Ln_3GaO_6{:}H}_i^q) - E(\mathrm{Ln_3GaO_6}) - \mu_{\mathrm{H}} + q(E_{\mathrm{HOMO}} + E_{Fermi}) + \Delta E_{\mathrm{LZ}}, \quad \text{(S1)}$$

where $E(\mathrm{Ln_3GaO_6{:}H}_i^q)$ is the energy of a supercell including a H_i with charge q and μ_H is the chemical potential of H. The notations of the other variables are the same as equation 1. The μ_H value at 0 K depends on the source gas: the conditions from the source gas of H_2 (H-rich) and from the source gas of H_2O (H-poor) are obtained by ½$E(H_2)$ and ½$E(H_2O)$ − ¼E(O_2), respectively. The dependences of μ_H on pressure and temperature can be calculated using the thermodynamic table [3, 7] and modified form for H_2 and H_2O from equation 5.

Supplementary Figure S10 shows E_H as a function of the Fermi level of La_3GaO_6. When the Fermi level is near the LUMO, H^- is stabilized, whereas when the Fermi level is at the band gap center or near the HOMO, H^+ is stabilized. The range of the Fermi level for stabilizing H^0 is not found. Because we are interested in the case where acceptor dopants substitute for Ln^{3+} or Ga^{3+} introducing negatively charged defects, we focus on the case where the Fermi level is shifted to the HOMO and positively charged defects are necessary to compensate for the charge imbalance.

When the Fermi level is close to the HOMO (doped case with acceptor dopants), the temperature is 0 K, and the pressure is 1 atm, E_H values of H^+ under the H-rich and H-poor conditions are both negative, which means that H^+ is spontaneously incorporated in La_3GaO_6. Then, E_H values of H^+ increase with

increasing temperature. When the temperature are 1000 K (temperature for application) and 1500 K (sintering temperature), under the H-rich condition, E_H value of H^+ keeps the negative value, whereas under the H-poor condition, E_H value of H^+ becomes positive value. This suggests that the incorporation of H^+ depends on the environment such as the source gas and temperature. This is also in agreement with the experimental report that a portion of proton conductivity decreases with increasing temperature [4].

Supplementary Figure S10 also shows E_v as a function of the Fermi level of La_3GaO_6. When the temperature and pressure are 0 K and 1 atm, respectively, E_v value is much higher than E_H value regardless of source gas of H. In contrast to the increase of E_H value, the E_v value decreases with increasing temperature. When temperature is 1000 K and Fermi level is near the HOMO, the region for E_v value lower than E_H value is found under the H-poor condition. When temperature is 1500 K, E_v value becomes lower than E_H value when the Fermi level slightly moves toward HOMO under the H-poor condition. In addition, the region for E_v value lower than E_H value is found even under the H-rich condition. The E_v value decreases with decreasing the pressure of O_2. The E_H value increases with decreasing the pressure of H_2O. When temperature is 1500 K at the lower pressure of 10^{-6} atm, the E_v value is already lower than the E_H value when the Fermi level is at the band gap center. This implies that the dominant defect between V_O^{2+} and H^+ depends on the environmental variables.

The other Ln_3GaO_6 also showed a similar trend for low E_H value of H^+ as shown in Supplementary Figure S11. As atomic numbers of Ln increase, the E_H values of H^+ increase; therefore, the incorporation of H^+ is easier for Ln_3GaO_6 with earlier Ln. When the Fermi level is close to the HOMO and temperature is 1000 K, under the H-rich condition, the eight Ln_3GaO_6 have all negative E_H value of H^+, whereas under the H-poor condition, they have all positive E_H value of H^+. This suggests that not only Gd_3GaO_6 but also other Ln_3GaO_6 may have proton conductivity, and the stability of H^+ depends on the environment.

When we introduce negatively charged defects by substituting acceptor dopants for Ln^{3+} or Ga^{3+}, generation of positively charged defects (H^+ and V_O^{2+}) may compete with each other to compensate for the charge imbalance. Therefore, we need to suppress the incorporation of H^+ to prevent interruption of V_O^{2+} generation for a suitable application of oxygen-ion conductor. Increasing temperature or decreasing oxygen gas and water vapor pressures can lead to increase of generation of V_O^{2+} (decrease of E_v) and decrease of incorporation of protons (increase of E_H).

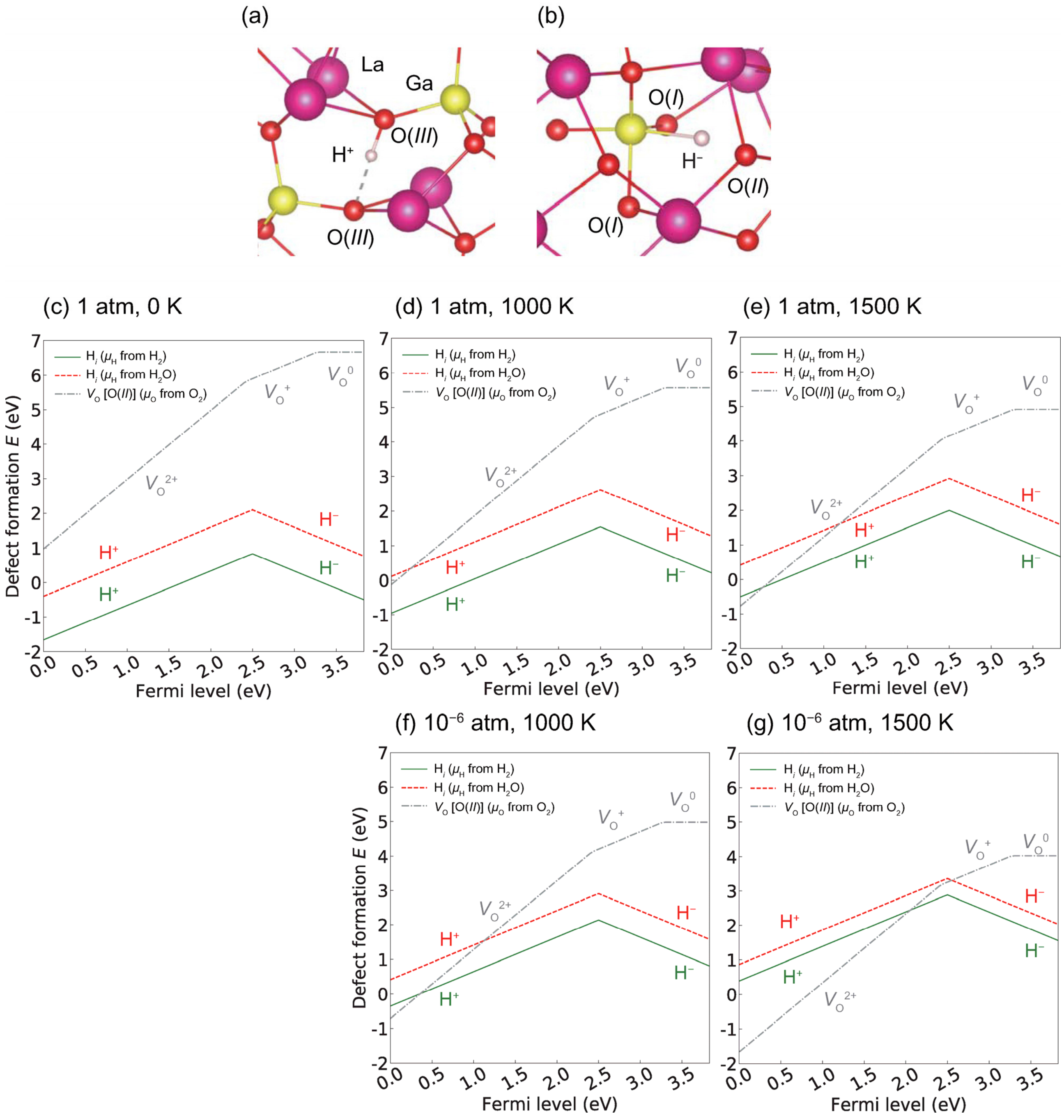

Figure S10. Stable sites for (a) H^+ and (b) H^- in La_3GaO_6. Defect formation energies (E_v and E_H) as a function of Fermi level of La_3GaO_6 when pressures of each gas and temperatures are (c) 1 atm and 0 K, (d) 1 atm and 1000 K, (e) 1 atm and 1500 K, (f) 10^{-6} atm and 1000 K, and (g) 10^{-6} atm and 1500 K, respectively. E_v is obtained from the O(*II*) site as this site is the most favorable for V_O^{2+}. The gradient of the lines denotes the charge of the defect.

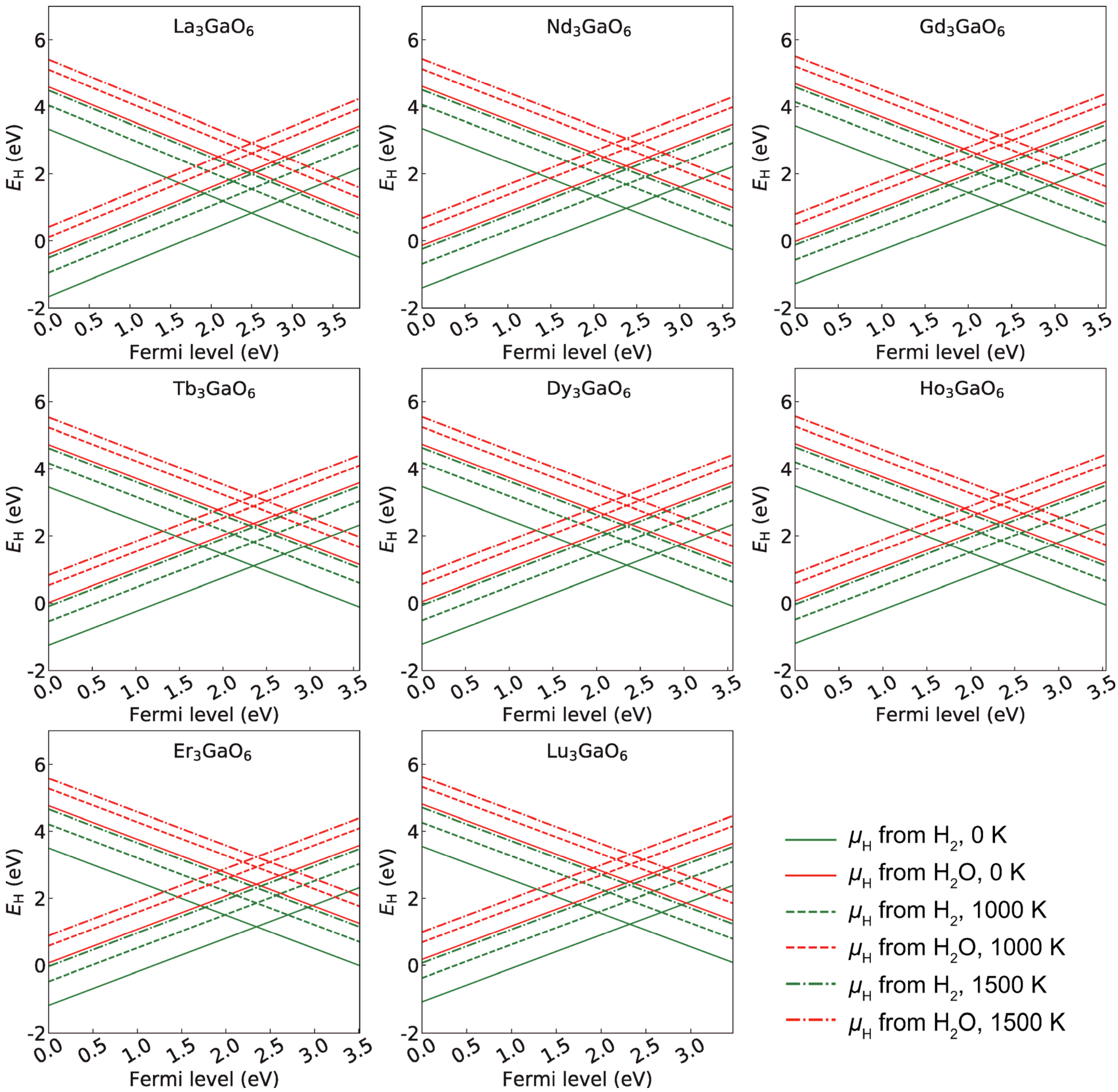

Figure S11. E_H as a function of Fermi level of eight Ln_3GaO_6 (Ln = La, Nd, Gd, Tb, Ho, Dy, Er, or Lu) when the pressure is 1 atm. The gradient of the lines denotes the charge of the defect.

References

[1] Liu F, Liu Q, Liang J, et al. A systematic study on crystal structure and magnetic properties of Ln_3GaO_6 (Ln= Nd, Sm, Eu, Gd, Tb, Dy, Ho and Er). J Solid State Chem. 177;2004:1796–1802.

[2] Greenwood NN, Earnshaw A. Chemistry of the Elements. Elsevier ; 2012.

[3] Stull DR, Prophet H, JANAF thermochemical tables, National Standard Reference Data System ; 1971.

[4] Iakovleva A, Chesnaud A, Animitsa I, et al. Insight into the synthesis and electrical properties of alkali-earth-substituted Gd_3GaO_6 oxide-ion and proton conductors. Int J Hydrog Energy. 41;2016:14941–14951.

[5] Kang Y, Han S. An origin of unintentional doping in transition metal dichalcogenides: the role of hydrogen impurities. Nanoscale. 9;2017:4265–4271.

[6] Yim K, Youn Y, Lee M, et al. Computational discovery of p-type transparent oxide semiconductors using hydrogen descriptor. npj Comput Mater. 4;2018:17.

[7] Reuter K, Scheffler M. Composition, structure, and stability of RuO_2 (110) as a function of oxygen pressure. Phys Rev B. 65;2001:035406.
